# Phenotypic variation of transcriptomic cell types in mouse motor cortex

**DOI:** 10.1038/s41586-020-2907-3

**Published:** 2020-11-12

**Authors:** Federico Scala, Dmitry Kobak, Matteo Bernabucci, Yves Bernaerts, Cathryn René Cadwell, Jesus Ramon Castro, Leonard Hartmanis, Xiaolong Jiang, Sophie Laturnus, Elanine Miranda, Shalaka Mulherkar, Zheng Huan Tan, Zizhen Yao, Hongkui Zeng, Rickard Sandberg, Philipp Berens, Andreas S. Tolias

**Affiliations:** 1 Center for Neuroscience and Artificial Intelligence, Baylor College of Medicine, Houston, TX USA; 2 Department of Neuroscience, Baylor College of Medicine, Houston, TX USA; 3 Institute for Ophthalmic Research, University of Tübingen, Tübingen, Germany; 4 International Max Planck Research School for Intelligent Systems, Tübingen, Germany; 5 Department of Pathology, University of California San Francisco, San Francisco, CA USA; 6 Department of Cell and Molecular Biology, Karolinska Institutet, Stockholm, Sweden; 7 Jan and Dan Duncan Neurological Research Institute, Houston, TX USA; 8 Allen Institute for Brain Science, Seattle, WA USA; 9 Center for Integrative Neuroscience, University of Tübingen, Tübingen, Germany; 10 Institute for Bioinformatics and Medical Informatics, University of Tübingen, Tübingen, Germany; 11 Bernstein Center for Computational Neuroscience, University of Tübingen, Tübingen, Germany

**Keywords:** Cellular neuroscience, Genetics of the nervous system

## Abstract

Cortical neurons exhibit extreme diversity in gene expression as well as in morphological and electrophysiological properties^1,2^. Most existing neural taxonomies are based on either transcriptomic^3,4^ or morpho-electric^5,6^ criteria, as it has been technically challenging to study both aspects of neuronal diversity in the same set of cells^7^. Here we used Patch-seq^8^ to combine patch-clamp recording, biocytin staining, and single-cell RNA sequencing of more than 1,300 neurons in adult mouse primary motor cortex, providing a morpho-electric annotation of almost all transcriptomically defined neural cell types. We found that, although broad families of transcriptomic types (those expressing *Vip*, *Pvalb*, *Sst* and so on) had distinct and essentially non-overlapping morpho-electric phenotypes, individual transcriptomic types within the same family were not well separated in the morpho-electric space. Instead, there was a continuum of variability in morphology and electrophysiology, with neighbouring transcriptomic cell types showing similar morpho-electric features, often without clear boundaries between them. Our results suggest that neuronal types in the neocortex do not always form discrete entities. Instead, neurons form a hierarchy that consists of distinct non-overlapping branches at the level of families, but can form continuous and correlated transcriptomic and morpho-electrical landscapes within families.

## Abstract (web summary)

Single-cell transcriptomic, morphological and electrophysiological characteristics are combined to classify more than 1,300 neurons from mouse motor cortex.

## Main

As animals can be grouped into species and assembled into a hierarchy of phylogenetic relationships to form the ‘tree of life’, neurons in the brain are thought to form discrete cell types, which in turn can be cast in a hierarchy of neuronal families and classes. The current view is that a neuronal cell type is characterized by a common genetic profile that gives rise to distinct physiological and anatomical properties, including patterns of connectivity^7,9^. A comprehensive multi-modal taxonomy of neurons would resemble a ‘parts list’ of the brain, helping us to decipher its bewildering complexity^10,11^.

For decades, neurons have been classified into types by their anatomical and physiological characteristics, and more recently by molecular markers^1,2,12,13^. High-throughput single-cell sequencing techniques have identified dozens of types of neuron on the basis of their transcriptional profiles^3,4,14,15^, but linking transcriptomically defined cell types (t-types) to their phenotypes has remained a major challenge^16^. However, to understand the roles of t-types in cortical computations, it is necessary to know their anatomy, connectivity, and electrophysiology^7^.

Our work is part of the BRAIN initiative cell census network (BICCN) effort to fully characterize the cellular taxonomy of neurons in mouse primary motor cortex (MOp). We used the Patch-seq technique^8,17–19^ to describe the morpho-electric phenotypes for most of the t-types in MOp^20^. Our analysis suggests that, in both excitatory and inhibitory classes of neurons, broad transcriptomic families (also known as ‘subclasses’^20^) have largely distinct phenotypes, but uncovers continuous morpho-electric variation within most of these families.

## Patch-seq of mouse primary motor cortex

**Extended Data Fig. 1:**
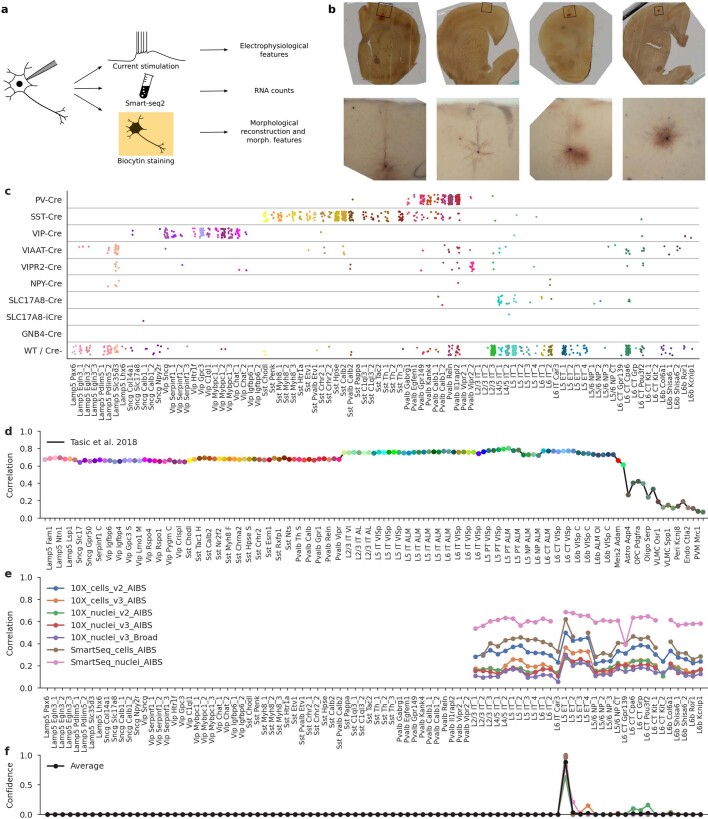
Patch-seq protocol, mouse Cre lines, and t-type assignment. **a**, Patch-seq combines electrophyiological recordings, RNA sequencing using Smart-seq2, and biocytin staining in the same cell. **b**, Four exemplary slice images. Top: an image of the whole slice using 4x magnification. Bottom: a flattened 3D image stack using 20× magnification. From left to right: L5 ET neuron, L2/3 IT neuron, L5 *Sst* neuron, L5 *Pvalb* neuron. **c**, t-Types assigned to cells collected in mice from different Cre lines. ‘WT/Cre-’ stands for cells from any Cre line that were not labelled with a fluorescent indicator, or for the cells patched in wild type mice. 1,227 cells shown. **d**, t-Type assignment procedure for one example cell (**d**–**f**). Correlations to the mean log expression of all t-types from ref. ^4^, using 3,000 most variable genes. Maximum correlation is to the excitatory neurons. t-Type names are shortened, and every second one is omitted for compactness. **e**, Correlations to all excitatory t-types from ref. ^20^ using all seven reference data sets and 500 most variable genes. **f**, t-Type assignment confidences for all seven data sets, obtained via bootstrapping over genes. The average confidence is shown in black. The mode of the average confidence was taken as the final t-type.

**Extended Data Fig. 2:**
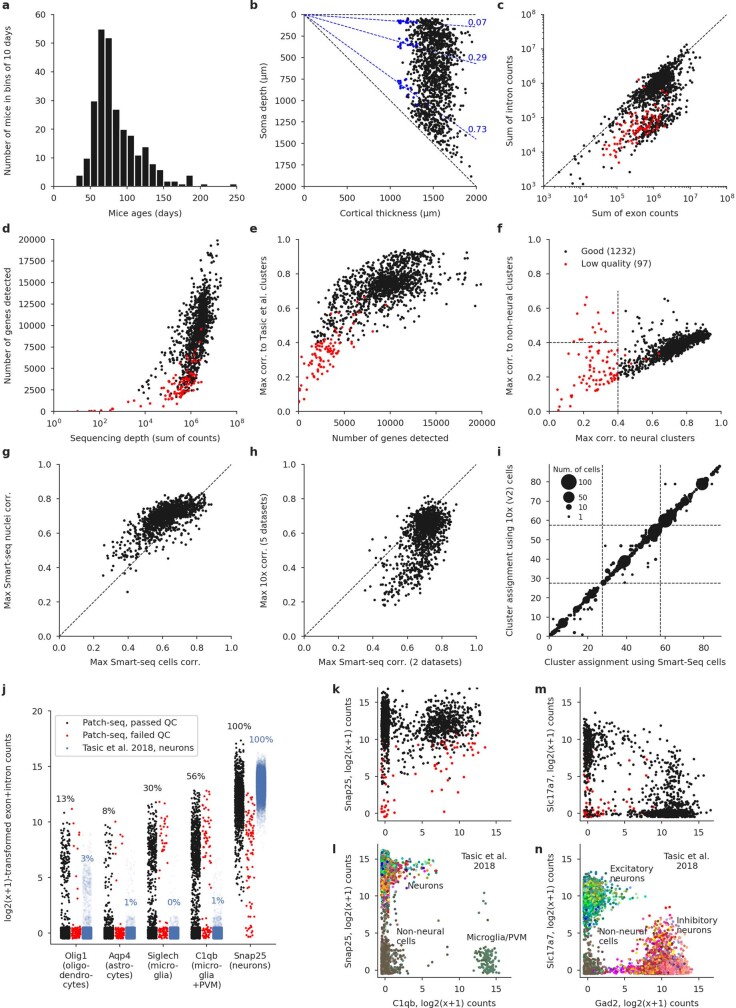
Quality control. **a**, Age distribution of the mice used in the experiments. Median: 75 days. **b**, Soma depths of all cells and cortical thickness of the corresponding slices. Dashed lines show layer boundaries, based on the Nissl-stained slices (measured layer boundaries shown as blue points). All soma depths were normalized by dividing them by the cortical thickness. **c**, Relationship between the number of exonic and intronic counts. The apparent bimodality could be explained by whether the nucleus was extracted or not during Patch-seq aspiration. Whenever the nucleus was not extracted, low amount of nonspliced RNA led to low intronic counts; otherwise, the number of intronic and exonic counts was almost the same. Red: cells eventually failing quality control. **d**, Relationship between sequencing depth (total number of reads) and the number of detected genes (number of genes with non-zero counts). **e**, Relationship between the number of detected genes and the maximal correlation to clusters from ref. ^4^. Cells with maximal correlation below 0.4 were declared low quality. **f**, Relationship between the maximal correlation across neural clusters and the maximal correlation across non-neural clusters from ref. ^4^. Cells with maximal neural correlation below 0.4 were declared low quality. See Methods for additional QC criteria. **g**, Maximal correlations using single-cell and single-nucleus Smart-seq2 reference data sets^20^. **h**, Maximal correlations using Smart-seq2 reference data sets (maximum across cell types and across two data sets) and using 10x reference data sets (maximum across cell types and across five data sets). **i**, t-Type assignment using single-cell Smart-seq2 reference data set and using single-cell 10x v2 reference data set. All points are on the integer grid; marker size shows the number of cells at the corresponding location. Dashed lines separate CGE-derived interneurons, MGE-derived interneurons, and excitatory neurons. The mapping was done within each order, so there cannot be any cells outside of the diagonal blocks. **j**, Expression of several prominent markers of non-neural cells, in comparison to the Smart-seq2 data set from ref. ^4^. The values are log_2_(*x* + 1)-transformed sums of exonic and intronic counts, shown with random $U\left(-\frac{1}{2},\frac{1}{2}\right)$ jitter. Percentage values refer to the fraction of cells with non-zero counts. PVM stands for perivascular macrophages. We selected these markers because they have very low expression in neural cells. A neuronal marker *Snap25* is shown for comparison. Cells from the reference data set are shown with the alpha-level set to the ratio of our data set size to that data set size (0.06), to make the dot plots more comparable. **k**, **l**, Neural and glial expression in our data set (**k**) and in the FACS-sorted data set^4^ (**l**) (plotted using the colours from the original publication, without transparency). **m**, **n**, The same using the excitatory marker *Slc17a7* and the inhibitory marker *Gad2*.

We sampled neurons across all layers (L) of adult mouse MOp (median postnatal day (P) 75) using various Cre driver lines to cover as diverse a population of neurons as possible. Neurons in acute slices were patch-clamped and stimulated with brief current pulses to record their electrophysiological activity at room temperature and then filled with biocytin for subsequent morphological recovery and reconstruction, and their RNA was extracted and sequenced using the Smart-seq2 protocol^21^ (Extended Data Fig. 1). In total, we performed whole-cell recordings from more than 2,000 cells, of which 1,329 cells (from 266 mice) passed initial quality control. The mRNA of these 1,329 cells was sequenced, yielding on average 1.3 million exonic and 0.7 million intronic reads (medians; mean ± s.d. on a log_10_ scale: 6.0 ± 0.6 and 5.6 ± 0.8, respectively) and 9,100 ± 3,500 (mean ± s.d.) detected genes per cell (Extended Data Fig. 2). Of these neurons, 646 had sufficient staining for their morphologies to be reconstructed.

Using the gene expression profiles, we mapped all sequenced neurons to the transcriptomic cell types (t-types) that have been identified using dissociated cells in a parallel study within the BICCN consortium^20^. To assign cell types, we used a nearest centroid classifier with Pearson correlation of log-expression across the most variable genes as a distance metric (Extended Data Fig. 1). Bootstrapping over genes was used to assess mapping confidence. The mapping was done separately using each of the seven reference data sets obtained with different sequencing technologies, including single-cell and single-nucleus Smart-seq2 and 10x sequencing^20^. We found that Patch-seq expression profiles were most similar to the single-nucleus Smart-seq2 data (Extended Data Fig. 2g, h). At the same time, there was good agreement between t-type assignments based on Smart-seq2 and those based on 10x reference data (Extended Data Fig. 2i), so consensus t-type assignment over all seven reference data sets was used for all subsequent analysis. Cells that showed poor mapping (owing to a low read count or excessive RNA contamination) were excluded (Extended Data Fig. 2), leaving 1,227 neurons for further analysis (817 inhibitory, 410 excitatory; 369 and 269 with morphological reconstructions, respectively).

**Fig. 1:**
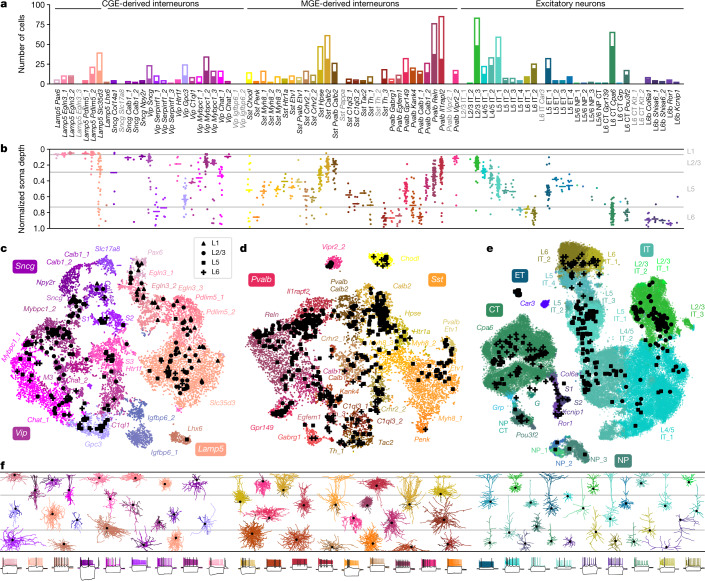
Transcriptomic coverage. **a**, Number of Patch-seq cells assigned to each of the neural transcriptomic types (t-types)^20^. Colours and the order of types are taken from the original publication^20^. The filled part of each bar shows the number of morphologically reconstructed neurons. Grey labels, t-types with no cells. Total number of neurons, 1,227. **b**, Normalized soma depths of all neurons of each t-type. For t-types with at least three cells, horizontal lines show medians. Soma depths were normalized by the cortical thickness in each slice (0, pia; 1, white matter). Grey horizontal lines, approximate layer boundaries identified by Nissl staining (L1, 0.07; L2/3, 0.29; L5, 0.73). Total number of neurons, 1,187 (for some cells soma depth could not be measured owing to failed staining). **c**, t-SNE representation of CGE-derived interneurons from the single-cell 10x v2 reference data set (*n* = 15,511; perplexity, 30). t-Type names are shortened by omitting the first word; some are abbreviated. Patch-seq cells from the *Vip*, *Sncg*, and *Lamp5* subclasses were positioned on this t-SNE atlas^23^ (black symbols). **d**, As in **c** but for MGE-derived interneurons (*n* = 12,083; perplexity, 30). **e**, As in **c** but for excitatory neurons (*n* = 93,829; perplexity, 100). **f**, Example morphologies coloured by t-type. For interneurons, dendrites are shown in darker colours. For excitatory neurons, only dendrites are shown. Black dots mark soma locations. Three voltage traces are shown below for some exemplary cells: the hyperpolarization trace obtained with the smallest current stimulation, the first depolarization trace that elicited at least one action potential, and the depolarization trace showing maximal firing rate. Stimulation length, 600 ms.

The resulting data set covered 77 out of the 90 neuronal t-types (Fig. 1a), with 73 t-types having at least one morphologically reconstructed neuron. The coverage was good for interneurons derived from the caudal and medial ganglionic eminences (CGE and MGE) and for excitatory neurons. Within-type distributions of soma depths (Fig. 1b) agreed well with previous data^4^ and with the layer-specific nomenclature of excitatory t-types, confirming the validity of our t-type assignment. Positioning all cells on reference maps made with *t*-distributed stochastic neighbour embedding (t-SNE)^22,23^ also showed good overall coverage (Fig. 1c–e) with only few uncovered regions.

**Extended Data Fig. 3:**
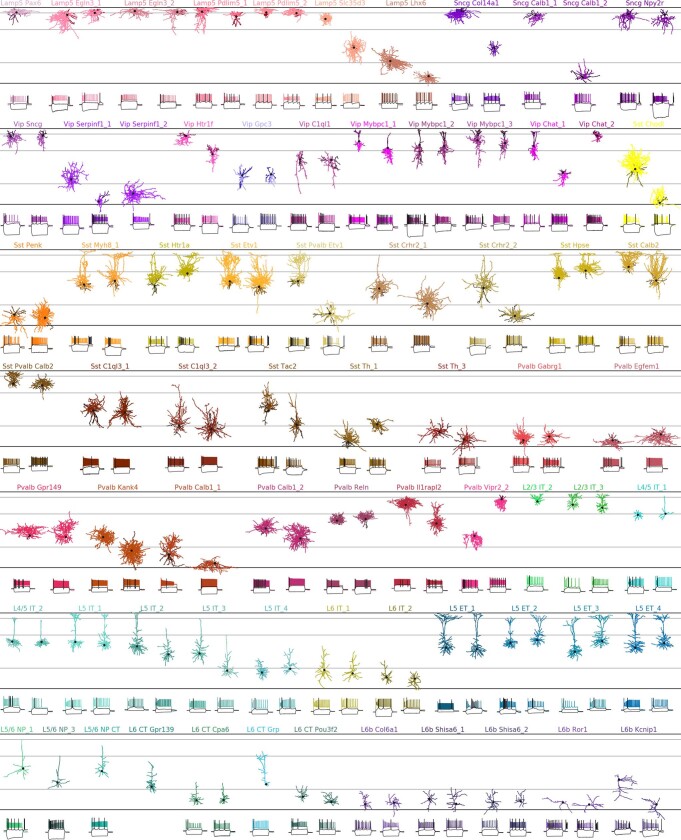
Diversity of mouse cortical neurons. Two representative examples per t-type, or one if only one reconstruction was available. In total 135 neurons in 73 t-types. For interneurons, dendrites are shown in darker colours. For excitatory neurons, only dendrites are shown. Black dots mark soma locations. Horizontal grey lines show approximate layer boundaries. Three voltage traces are shown for each neuron: the hyperpolarization trace obtained with the smallest current stimulation, the first depolarization trace eliciting at least one action potential, and the depolarization trace showing maximal firing rate. Stimulation length: 600 ms. The length of the shown voltage traces: 900 ms. Electrophysiological recording for one neuron did not pass quality control and is not shown.

The observed phenotypes included most of the morphological and electrophysiological types of cortical neurons that have been described previously in mice and rats^5,6,24^, allowing us to link transcriptomic and morpho-electric descriptions (Extended Data Fig. 3, Supplementary File 1).

A detailed description of all t-types is provided in Extended Data Tables 1, 2. One interesting case was the transcriptomically isolated *Lamp5 Lhx6* type, which consists of deep L5/L6 neurogliaform cells (NGCs). This type, unlike all other *Lamp5* types, is putatively MGE-derived^4^, so its identity was an open question^16^. Our results suggest that although all deep NGCs belong to the *Lamp5* subclass, some are derived from the CGE and some from the MGE, as in the hippocampus^25–27^. Another finding was that the *Sst Pvalb Calb2* type, which is transcriptomically in between the *Sst* and *Pvalb* subclasses, was also in between these subclasses in terms of its morpho-electric phenotype^28^. Furthermore, we confirmed that chandelier cells from both superficial and deep layers belonged to transcriptomically isolated *Pvalb Vipr2* types. We also showed that three previously described morphological types of L5 *Pvalb* cells^5^, as well as two morphological types of L5 Martinotti cells^29,30^, corresponded to different t-types. We were also able to identify a t-type, L4/5 IT_1, that was located on the boundary between L2/3 and L5 and probably corresponds to the quasi-L4 neurons described previously in motor cortex^31^.

## Distinct phenotypes of major families

**Fig. 2:**
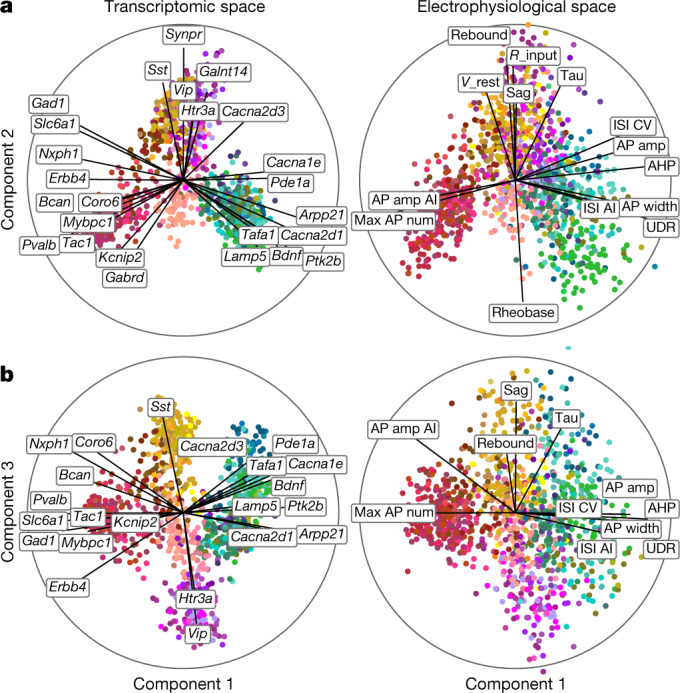
Sparse reduced-rank regression. **a**, **b**, A sparse reduced-rank regression (RRR) model^32^ to predict combined electrophysiological features from gene expression. Transcriptomic data are linearly projected to a low-dimensional space that allows reconstruction of electrophysiological data; components 1 and 2 (**a**) and 1 and 3 (**b**) of rank-5 model are shown. *n* = 1,219. Colour corresponds to t-type. The model selected 25 genes (left). Each panel is a biplot, showing correlations of original features with both components; the circle corresponds to correlation 1. Only features with average correlation above 0.4 are shown. Labels were automatically positioned to reduce overlap. AI, adaptation index; AP, action potential; CV, coefficient of variation; ISI, interspike interval; *R*_input, input resistance; *V*_rest, resting potential; UDR, upstroke-to-downstroke ratio.

**Extended Data Fig. 4:**
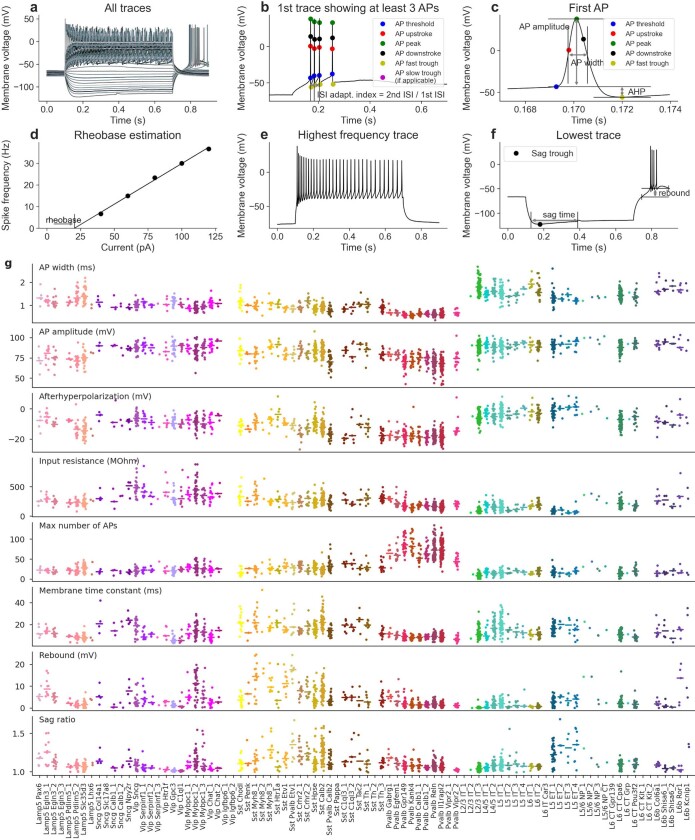
Extraction and distribution of electrophysiological features. Panels **a**–**f** show data from the same exemplary cell. **a**, Membrane potential responses to the consecutive step current injections. Hyperpolarizing currents were used to compute the input resistance (274.80 MOhm) and membrane time constant tau (21.95 ms). **b**, The first five traces showing spikes were used to compute ISI adaptation index (1.26), ISI average adaptation index (1.15), AP amplitude adaptation index (0.91) and AP amplitude average adaptation index (0.99). **c**, The first AP elicited in this neuron. It was used to compute AP threshold (−40.18 mV), AP amplitude (81.17 mV), AP width (0.80 ms), AHP (−12.60 mV), ADP (0 mV), UDR (1.62) and latency of the first spike (69.28 ms). **d**, Regression line gives the rheobase estimate (20.44 pA). **e**, The highest firing trace with 32 APs. This trace was used to estimate the ISI CV (0.27), ISI Fano factor (0.0014 ms), AP CV (0.17) and AP Fano factor (1.32 mV). **f**, The lowest hyperpolarization trace was used to compute the sag ratio (1.17), sag time (0.26 ms), sag area (31.16 mV⋅ms) and rebound (17.84 mV). **g**, Eight important electrophysiological features are shown for all cells across all t-types. For t-types with at least three cells, horizontal lines show median values. See Supplementary File 2 for all electrophysiological features.

**Extended Data Fig. 5:**
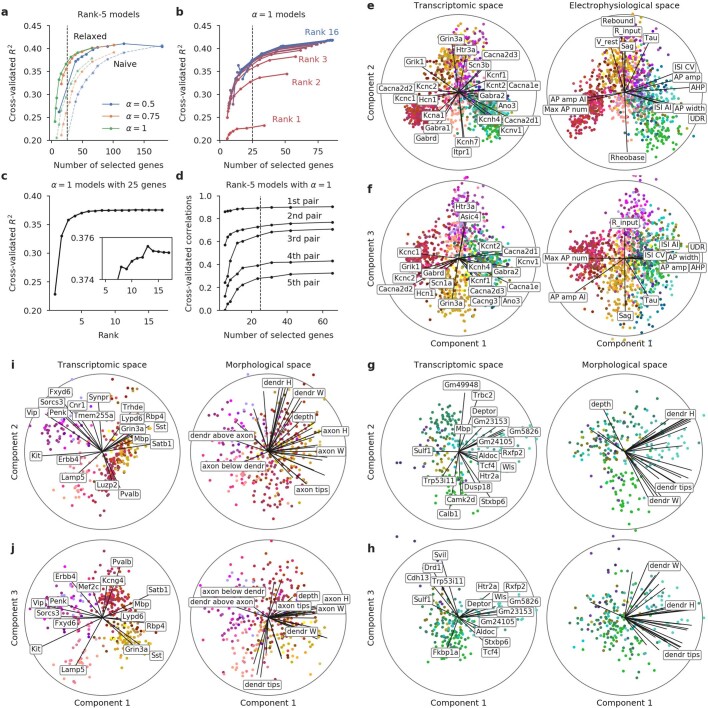
Additional reduced-rank regression analysis and cross-validation. **a**, Cross-validated *R*^2^ of ‘naive’ and ‘relaxed’ sparse RRR solutions^32^ for various elastic net penalties (*α* and *λ*). ‘Relaxed’ means that the model was re-fit without a lasso penalty using only the selected genes; ‘naive’ means that it was not re-fit. Vertical dashed lines at 25 genes corresponds to the choice made for Fig. 2. The best performance is around ~100 genes, but we chose 25 for the sake of interpretability. The subsequent panels only show results for the ‘relaxed’ models. **b**, Cross-validated *R*^2^ using *α* = 1 for different ranks from rank 1 to rank 16 (full rank). **c**, Cross-validated *R*^2^ using *α* = 1 and *λ* needed to obtain 25 genes for different ranks. The peak performance is achieved with rank ~13 (inset), but rank-5 model used in the main text is almost as good. **d**, Cross-validated correlations between sequential projections of the transcriptomic and electrophysiological data sets (rank-5 models with *α* = 1). For any given number of selected genes, correlations decrease monotonically for higher components. **e**, **f**, Reduced-rank regression model using only ion channel genes. A full analogue of Fig. 2 but using only 328 ion channel genes (see Methods), of which 307 were detected in our data set in at least 10 cells. **g**–**j**, Reduced-rank regression model predicting morphological features. An analogue of Fig. 2 but using morphological, instead of electrophysiological features. The analysis was done separately for the excitatory (g–h) and for the inhibitory (i–j) neurons because different sets of morphological features were computed for these sets of neurons. Excitatory neurons: 269 cells, 35 features. Rank-5 model, *λ* = 0.59, adjusted to yield 25 genes. Only a subset of morphological features are labelled to reduce the clutter (abbreviations: “W” — width, “H” — height). Inhibitory neurons: 367 cells, 50 features, *λ* = 0.49.

We next asked to what extent the morpho-electric phenotype could be predicted by gene expression across the entire data set. To obtain quantitative characterizations of the morpho-electric phenotypes, we extracted 29 electrophysiological (Extended Data Fig. 4, Supplementary File 2) and about 50 morphological features for each cell. We first focused on 17 electrophysiological features and used sparse reduced-rank regression^32^, a technique that predicts the firing properties on the basis of a low-dimensional latent space representation computed from a sparse selection of genes. We used cross-validation to tune the regularization strength (Extended Data Fig. 5). The selected model used 25 genes with a 5-dimensional latent space and achieved a cross-validated *R*^2^ of 0.38. To visualize the structure of the latent space, we projected gene expression and electrophysiological properties onto the latent dimensions (Fig. 2). The cross-validated correlations between the first three pairs of projections were 0.90, 0.74, and 0.67, respectively.

These first three components clearly separated five major groups of neurons: the *Pvalb*, *Sst*, *Vip*, and *Lamp5* interneuron subclasses, and the excitatory neuron class (Fig. 2). These groups had distinct electrophysiological properties: for example, as expected, *Pvalb* neurons were characterized by high firing rates while *Sst* neurons had high values of the hyperpolarization sag and rebound (Fig. 2, right). Some of the genes selected by the model were prominent marker genes, such as the pan-inhibitory markers *Gad1* and *Slc6a1* (related to GABA (γ-aminobutyric acid) processing), or the more specific inhibitory markers *Sst*, *Vip*, *Pvalb*, *Tac1*, and *Htr3a*. Notably, some other selected genes were more directly related to electrophysiological properties, such as the calcium channel subunit genes *Cacna1e* and *Cacna2d3* or the potassium channel-interacting protein gene *Kcnip2*, which can modulate firing properties in individual families. A reduced-rank regression model restricted to using only ion channel genes (Extended Data Fig. 5) did not perform much worse than the full model (cross-validated *R*^2^ = 0.33 and correlations 0.86, 0.71, and 0.56, respectively, with regularization set to select 25 genes). Reduced-rank regression analysis using morphological features supported the separation of major families (Extended Data Fig. 5).

**Fig. 3:**
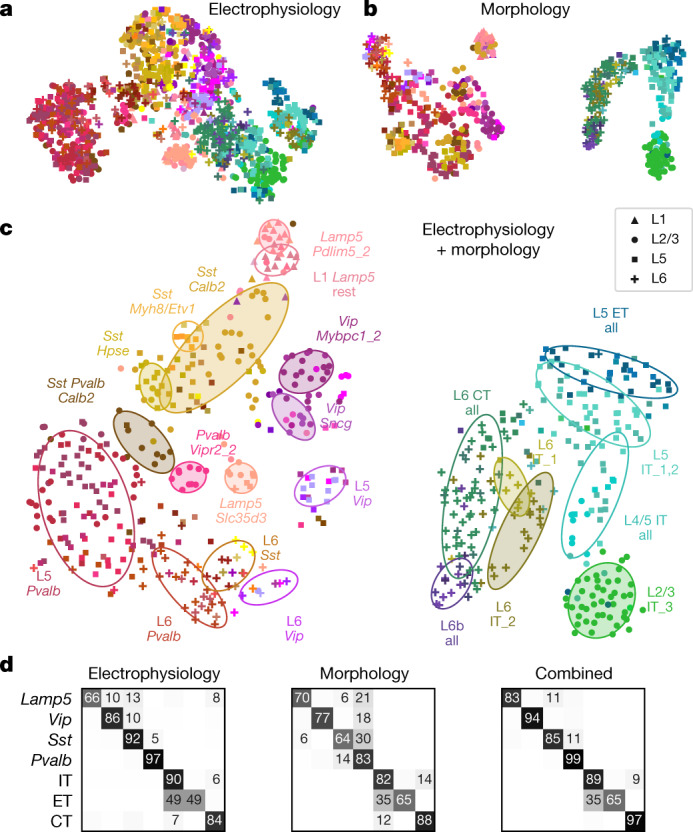
Morpho-electric t-SNE embeddings. **a**, t-SNE embedding constructed using electrophysiological features. Colour corresponds to t-type. *n* = 1,320 cells used to construct the embedding, 1,219 cells with t-type labels shown. Perplexity, 30. **b**, t-SNE embedding constructed using combined morphometric features and *z*-profiles. *n* = 636 cells. Perplexity, 30. **c**, t-SNE embedding constructed using combined electrophysiological and morphological features. *n* = 628 cells. Perplexity, 30. Ellipses show 80% coverage ellipses for the most prominent t-types (shaded) and for some groups of related t-types and some layer-restricted families (unshaded). We chose these groups to reduce the overlap between ellipses. **d**, Confusion matrices for classifying cells into seven transcriptomic families using kNN classifier (*k* = 10) and three feature sets. Each row shows what fraction of cells from a given family is classified in each of the seven families. The values in each row sum to 100% but only values above 5% are shown.

**Extended Data Fig. 6:**
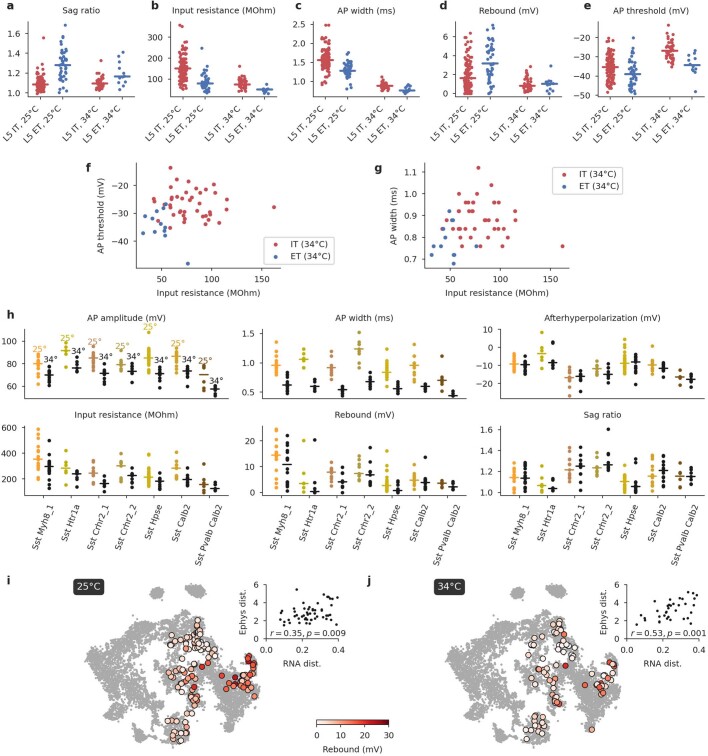
Electrophysiological properties of IT, ET, and *Sst* neurons in Layer 5 at physiological temperature. **a**–**e**, Each panel shows a comparison between L5 neurons from the IT and the ET subclasses (pooled across all t-types within each subclass). The main set of experiments was done at room temperature (25 °C). Follow-up experiments were done at physiological temperature (34 °C), in the presence of 1 mM kynurenic acid and 0.1 mM picrotoxin in order to block fast glutamatergic and GABAergic synaptic transmission. Horizontal lines show median values. The first four panels correspond to features showing the largest IT/ET differences at room temperature, according to the two-sided Wilcoxon-Mann–Whitney test statistic (and omitting several features that are very correlated with the shown ones: upstroke-to-downstroke ratio, sag time, and sag area). The last panel additionally shows one feature that showed prominent difference at 34 °C. **f**, **g**, IT and ET neurons recorded at 34 °C in two-dimensional representations using the features with highest separability. **h**, The change of electrophysiological properties between room temperature (25 °C) and physiological temperature (34 °C) for various t-types from the *Sst* subclass. Only L5 neurons are shown. Only t-types with ≥ 5 cells in both conditions are shown. Horizontal lines denote median values. AP amplitude and AP width changed the most between conditions, but the relative differences between t-types stayed roughly the same. The other four shown features did not change much, and the relative differences between t-types stayed the same. **i**, Overlay of the L5 *Sst* cells over the reference t-SNE embedding, coloured by rebound, as in Fig. 4b. The inset shows the correlation between transcriptomic distances and electrophysiological differences between all pairs of *Sst* t-types (only for t-types with at least 5 cells, and excluding *Sst Chodl*), together with its *p*-value. **j**, The same analysis as in (c) but using the experiments performed at physiological temperature. No corrections for multiple comparisons were applied.

Similarly, a 2D t-SNE embedding of Patch-seq cells based on the same electrophysiological features showed that the major transcriptomic families have distinct electrophysiological properties (Fig. 3a): the *Pvalb*, *Lamp5*, *Sst*, *Vip*, CT (corticothalamic), IT (intratelencephalic), and ET (extratelencephalic) subclasses were mostly well separated from each other. We quantified this separation using a confusion matrix for *k*-nearest neighbours (kNN) classification of cells into families: it was mostly diagonal, with only the ET and IT subclasses strongly overlapping (Fig. 3d). We confirmed the electrophysiological overlap between IT and ET neurons in follow-up experiments at 34 °C (Extended Data Fig. 6).

We also constructed a 2D t-SNE embedding based on the morphological features (Fig. 3b). We used only dendritic features for the excitatory cells, but both axonal and dendritic features for the inhibitory cells, leading to a strong separation between these two major classes. Within each class, cells were strongly segregated by the soma depth, with excitatory cells forming mostly a one-dimensional manifold. The separability between inhibitory families was weaker than with electrophysiological features (Fig. 3d). The between-family separability was strongest when we had combined electrophysiological and morphological features into a joint representation (Fig. 3c, d), showing that these sets of properties are not redundant. The ellipses in Fig. 3c highlight prominent t-types and groups of t-types with similar morpho-electric properties.

In summary, different transcriptomic families had largely distinct morpho-electric phenotypes, in agreement with them being well separated in the transcriptomic space^4^.

## Continuous phenotypic variation

**Fig. 4:**
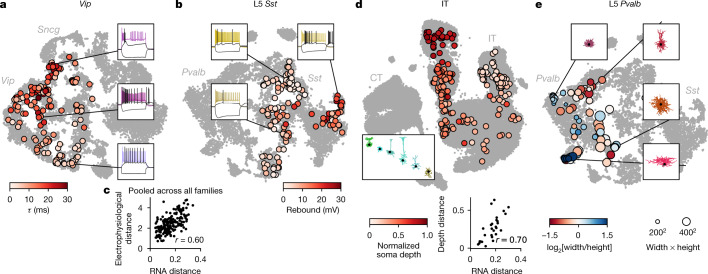
Phenotypic variability within transcriptomic families. **a**, *Vip* neurons mapped to the reference t-SNE embedding from Fig. 1c, coloured by membrane time constant (*τ*). Insets, example firing traces. **b**, *Sst* neurons from layer 5 (excluding *Sst Chodl* t-type) mapped to the reference t-SNE embedding from Fig. 1d, coloured by rebound value. **c**, Correlation between transcriptomic distances and electrophysiological distances across all 200 pairs of t-types from the same family (for 50 t-types with at least 5 cells), pooled across all families. Transcriptomic distance was computed using the reference 10x data as the correlation between average log-expression across most variable genes. Electrophysiological distance is Euclidean distance between the average feature vectors. **d**, IT neurons mapped to the reference t-SNE embedding from Fig. 1e, coloured by normalized soma depth. Inset, examples of IT neurons at different depths, coloured by t-type. Scatter plot used eight t-types with at least five cells and shows correlation between transcriptomic distances and cortical depth distances. Cortical depth distance is Euclidean distance between the average normalized soma depths. **e**, *Pvalb* neurons from layer 5 mapped to the reference t-SNE embedding from Fig. 1d, coloured by axonal width/height log-ratio. Circle area corresponds to the width × height product. Insets, example morphologies.

Within individual transcriptomic families, morpho-electric phenotypes rarely formed isolated clusters (Fig. 3). Moreover, we often found that morpho-electric phenotypes varied continuously from one t-type to another (Fig. 4). For example, electrophysiological properties of the t-types within the *Vip* subclass varied continuously across the transcriptomic landscape; the membrane time constant, for instance, had its largest values close to the *Sncg* subclass and gradually decreased towards *Vip Gpc3* (Fig. 4a). We observed the same in the *Sst* subclass, which is known to be transcriptomically^4^ and morpho-electrically^29,30,33^ diverse in L5. Here we also found that morpho-electric properties varied continuously across the transcriptomic landscape, with neighbouring t-types consistently showing similar morphologies and similar rebound values (Fig. 4b). We confirmed this effect in follow-up experiments at physiological temperature (Extended Data Fig. 6).

**Extended Data Fig. 7:**
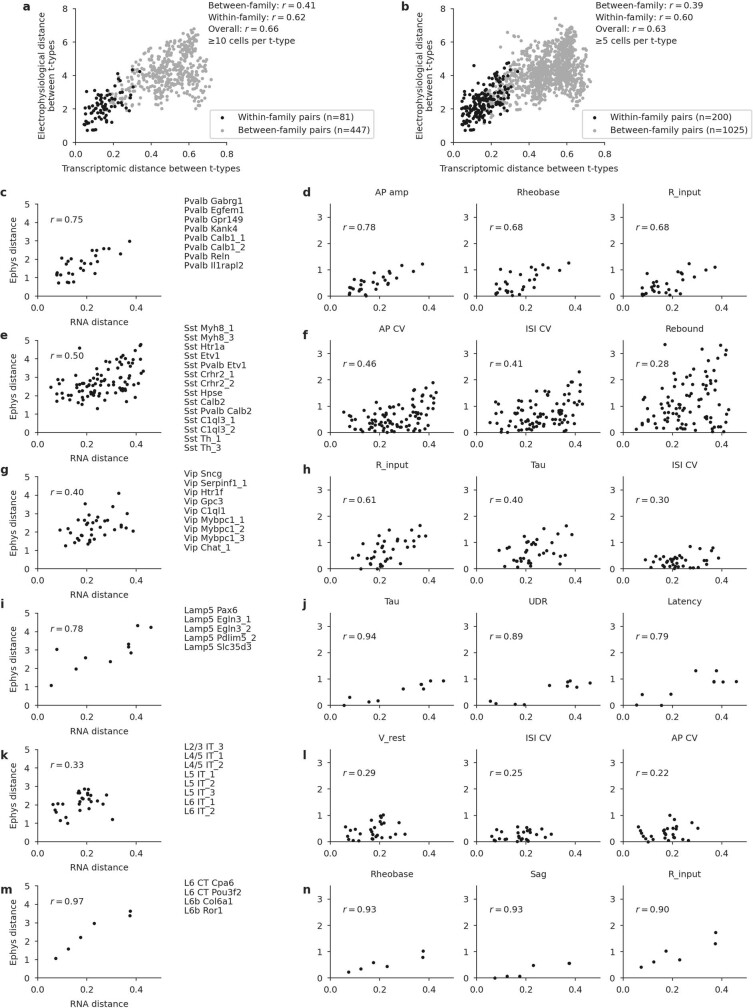
Transcriptomic and electrophysiological distances within individual families. **a**, **b**, Pooled within-family analysis. The same analysis as in Fig. 4c but showing within-family as well as between-family pairs of t-types. Using a cutoff of at least 10 neurons per t-type (a) and a cutoff of at least 5 neurons per t-type (b). **c**–**n**, Transcriptomic and electrophysiological distances within individual families. Only t-types with ≥ 5 neurons are used for this analysis (used t-types are listed in the second column). Transcriptomically well-isolated *Sst Chodl* and *Pvalb Vipr2_2* were excluded. Three electrophysiological features with the highest correlation to the transcriptomic distance are shown on the right, for each family.

To quantify this effect, for each pair of t-types within each family we computed the transcriptomic distance (correlation distance between average log-counts in the reference data) and the electrophysiological distance (Euclidean distance between average feature vectors) between them. Pooling the pairs across all families, we found that these two distance measures were correlated, with *r* = 0.60 (Fig. 4c, *n* = 200 pairs; Extended Data Fig. 7). The correlation was also observed within multiple individual families and for many individual electrophysiological features (Extended Data Fig. 7).

The IT subclass provides an example of a similar phenomenon in another data modality (Fig. 4d). IT neurons span all layers from L2/3 to L6, and IT t-types are largely layer-restricted^4^. However, we found that IT t-types did not form distinct groups for each cortical layer; instead, the soma depth and RNA expression varied continuously along a one-dimensional manifold (Fig. 4d), in agreement with parallel findings based on a spatial transcriptomics approach^34^. For example, L4/5 and L5 IT t-types that were transcriptomically close to the L2/3 IT t-types were located at the top of L5 close to the border between L2/3 and L5, whereas L5 IT t-types that were transcriptomically close to L6 IT t-types were located at the bottom of L5 close to the border with L6. Transcriptomic distances between t-types were strongly correlated with the average soma depth differences (*r* = 0.70; Fig. 4d).

Finally, the *Pvalb* subclass is usually understood as electrophysiologically homogenous (all neurons are fast spiking) but has been described as morphologically diverse, in particular in L5^5^. However, it was previously unclear whether different morphologies such as shrub-like or horizontally elongated correspond to different t-types^5^. While we found that different t-types had different preferred morphologies (Extended Data Table 1), they showed substantial overlap, in agreement with the L5 *Pvalb* t-types themselves not having clear boundaries^4^ (Fig. 1d). The shape of the axonal arbor showed continuous changes across the transcriptomic landscape (Fig. 4e): small shrub-like basket cells, horizontally elongated basket cells, and vertically elongated classical basket cells were located in different corners of the t-SNE embedding, with intermediate morphologies in between.

In summary, within major transcriptomic families, morpho-electric phenotypes and/or soma depth often varied smoothly across neighbouring t-types, indicating that transcriptomic neighbourhood relationships in many cases corresponded to similarities in other modalities.

## Variability in individual t-types

**Fig. 5:**
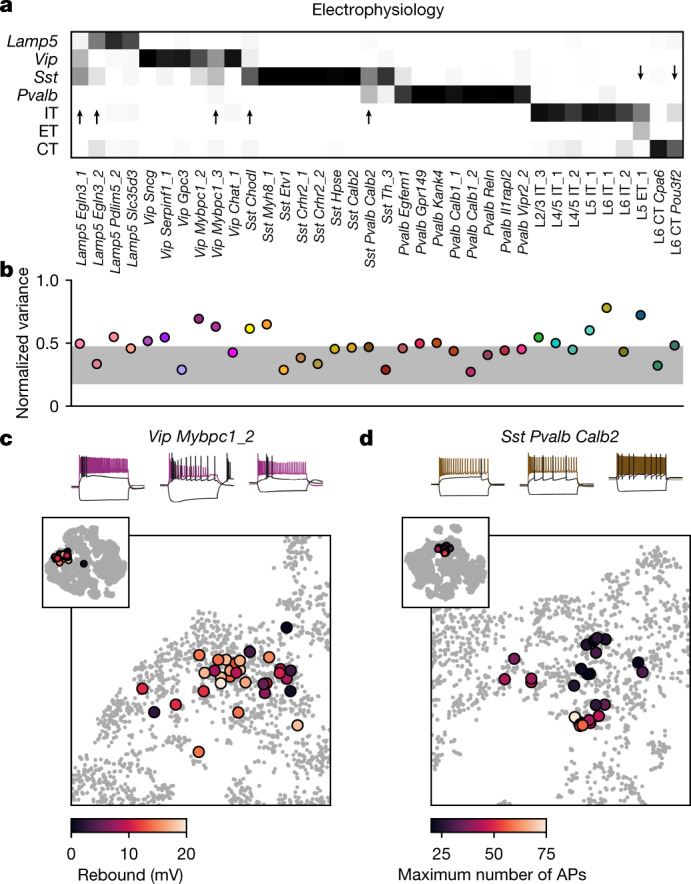
Phenotypic variability of individual t-types. **a**, Confusion matrix for classifying cells from each t-type into seven transcriptomic families using electrophysiological features. Only t-types with at least ten cells are shown. Values in each column sum to 1. Arrows mark t-types that are classified into wrong families more than 25% of the time. We used a kNN-based classifier with *k* = 10. **b**, Normalized total variance of features in each t-type. Higher values correspond to t-types with more variable phenotypes. Horizontal grey band, minimum to maximum normalized variances of *k*-means clusters. **c**, Three exemplary traces from *Vip Mybpc1_2* cells (all with confidence ≥ 95%) and t-SNE overlay coloured by rebound. Inset, the same t-SNE embedding as in Fig. 1. Main plot, magnification. **d**, Three exemplary traces from *Sst Pvalb Calb2* cells (confidence ≥ 95%) and t-SNE overlay coloured by maximum firing rate.

To study the morpho-electric phenotypes of individual t-types, we measured how consistently they conformed to their respective transcriptomic families (Fig. 5a) and how variable they were within a t-type (Fig. 5b). First, we used a kNN classifier to classify cells from each t-type with at least ten cells into transcriptomic families, using electrophysiological features. Most t-types could be unambiguously placed into the correct family (Fig. 5a), but some t-types were in between two families. For example, many *Sst Pvalb Calb2* neurons were classified as belonging to the *Pvalb* subclass on the basis of electrophysiology. Similarly, *Lamp5 Egln3_1* neurons had rather *Vip*- and *Sst*-like firing instead of the typical *Lamp5* electrophysiology, and *Vip Mybpc1* neurons often had *Sst*-like firing. Thus, while overall transcriptomic family was highly predictive of the cell phenotype, some t-types exhibited properties similar to those of another transcriptomic family.

Next, we measured the normalized total variance of each t-type using electrophysiological features and compared it to the normalized total variance of phenotype clusters derived by *k*-means clustering (with *k* set to the number of t-types). The rationale here was that the variance of the *k*-means clusters would reflect the minimal possible variance obtainable in our data set. Values much above the cluster variances indicate non-trivial phenotypic variability within a t-type.

**Extended Data Fig. 8:**
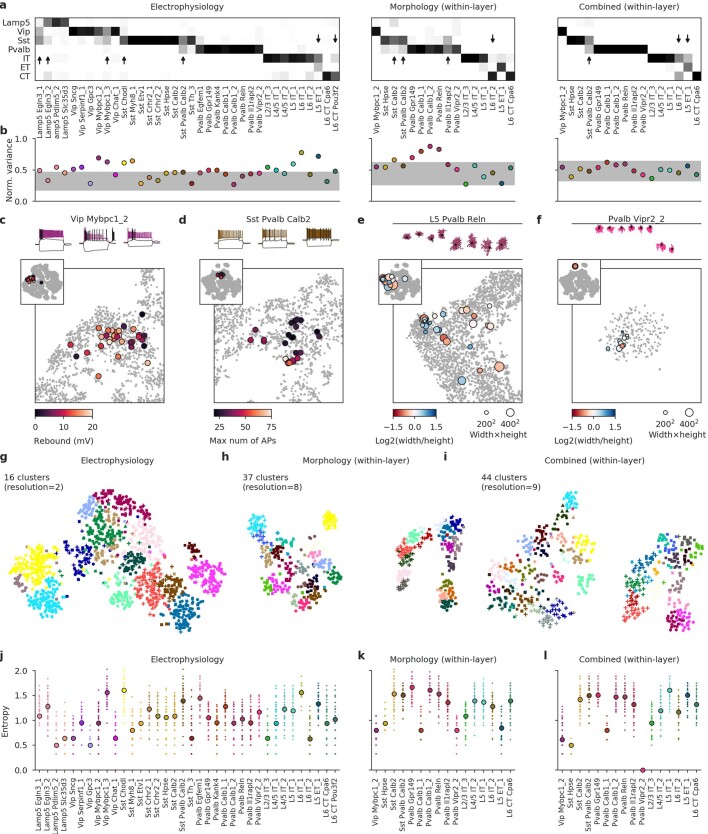
Phenotypic variability of individual t-types. The extended version of Fig. 5. **a**, Confusion matrices for classifying cells from each t-type into seven transcriptomic families, using electrophysiological, morphological, and combined features. Only t-types with at least 10 cells are shown. For morphological and combined features we only took cells from one cortical layer. Values in each column sum to 1. Arrows mark t-types that are classified into wrong families more often than 25% of the time. We used kNN-based classifier with *k* = 10. **b**, Normalized total variance of features in each t-type. Higher values correspond to t-types with more variable phenotypes. Horizontal grey band shows the min/max normalized variances of *k*-means clusters. **c**, Three exemplary traces of cells from the *Vip Mybpc1_2* type (all with confidence ≥ 95%) and t-SNE overlay coloured by the rebound. Inset: the same t-SNE embedding as in Fig. 1. Main plot: zoom-in. **d**, Three exemplary traces of cells from the *Sst Pvalb Calb2* (confidence ≥ 95%) and t-SNE overlay coloured by the maximum firing rate. **e**, Exemplary morphologies of L5 cells from the *Pvalb Reln* type and t-SNE overlay coloured by the axonal width/height log-ratio as in Fig. 4e. **f**, Exemplary morphologies of *Pvalb Vipr2_2* chandelier neurons and t-SNE overlay coloured by the axonal width/height log-ratio as in Fig. 4e. **g**–**i**, We used Leiden clustering^35^ to cluster the cells based on electrophysiological, morphological, and combined features. The clustering resolution was adjusted to roughly match the number of e-types, m-types, and em-types from ref. ^24^. The cluster colours in these panels are arbitrary and not the same as the colours used for t-types. **j**–**l**, For each t-type with at least 10 cells, we measured the entropy of the cluster assignments. Entropy zero corresponds to all cells getting into one cluster. Higher entropies mean that cells get distributed across many clusters. We repeated the clustering 100 times with different random seeds, and for each of them, subsampled each t-type to 10 cells to measure the entropy. Points show 100 repetitions, big markers show medians. When using morphological and combined features, all t-types were layer-restricted, as above. The t-type colours do not correspond to the colours in panels (**j**–**i**).

We found that many t-types had total variance substantially above the variances of the *k*-means clusters (Fig. 5b) and an alternative analysis using entropies of Leiden clustering^35^ often highlighted the same t-types as variable (Extended Data Fig. 8). Not all t-types showed high variability: some of them, such as *Pvalb Vipr2_2* (chandelier cells), appeared morpho-electrically homogeneous. By contrast, *Vip Mybpc1_2* was marked as having high electrophysiological variability and indeed had high variance in input resistance, membrane time constant, and rebound (Extended Data Fig. 4). This variability was not random: overlaying the rebound values on the t-SNE embedding (Fig. 5c) showed that cells with low rebound were located close to the boundary with the low-rebound *Vip Sncg* type. Similarly, *Sst Pvalb Calb2* cells had high variability in terms of the maximum firing rate, but high-firing cells were mostly grouped in one part of the transcriptomic landscape (Fig. 5d).

We found similar examples in the morphological modality (Extended Data Fig. 8). Together, these examples suggest that within-t-type morpho-electric variability can in some cases be related to the underlying transcriptomic variability. This is in agreement with the idea that on a fine within-family scale, both transcriptomic and morpho-electric landscapes are continuous rather than discrete.

## Discussion

We used Patch-seq to provide the missing link between transcriptomic and morpho-electric descriptions of neurons in adult mouse motor cortex. Broad transcriptomic families were mostly well separated in their morpho-electric properties. Previous studies using transgenic lines had shown that morpho-electric properties within these families can be highly variable^5,24^. We found that this variation is structured across the transcriptomic landscape, such that the morpho-electric distance between t-types within a family is correlated with their transcriptomic distance. Furthermore, we found non-trivial morpho-electric variability within multiple t-types. Although we cannot fully exclude the possibility that some of this variability can be attributed to technical challenges of Patch-seq or to factors such as the exact spatial location of the cell within motor cortex^36^, there are clear cases in our data that suggest that within-type morpho-electric variability is related to within-type transcriptomic variability.

We therefore suggest that the ‘tree of cortical cell types’ may look more like a banana tree with a few large leaves, rather than an olive tree with many small ones. In this metaphor, neurons follow a hierarchy consisting of distinct, non-overlapping branches at the level of families (large leaves), but with a spectrum of cells forming continuous and correlated transcriptomic and morpho-electrical landscapes within each leaf.

This is at odds with the notion that t-types are discrete entities, an implicit assumption behind any cluster analysis. Consistent with our interpretation, recent transcriptomic and anatomical studies have argued that neurons in hippocampus, striatum, and cerebellum can be better described as forming partially continuous manifolds^27,37–39^. Similarly, cortical studies have identified many intermediate cells with uncertain t-type assignments^3,4^. Thus, the goal to assemble an exhaustive inventory of neural cell types might be unattainable if the types, unlike the chemical elements in the periodic table, are not discrete entities. We believe that there is an urgent need for theoretical work on how to conceptualize and model hierarchical discrete/continuous cell variability in a principled way^7^.

Developmentally, it is thought that neural diversity is generated through a combination of intrinsic genetic programs in progenitor cells, and activity-dependent and environmental factors^40–44^. It remains unclear to what extent the interplay between hard-wired genetic programs and extrinsic cues might explain our observations.

Our study has several limitations. First, some t-types were covered only sparsely or not at all. Additional experiments with more specific Cre lines could fill some of the gaps, but some very rare putative t-types might not be amenable to Patch-seq study. Second, as the RNA extraction process may have interfered with biocytin diffusion^17^ and as MOp is quite thick, it was difficult to recover complete morphologies of some groups of neurons, such as deep L5 Martinotti cells with thin long axons that reach all the way to L1.

**Extended Data Fig. 9:**
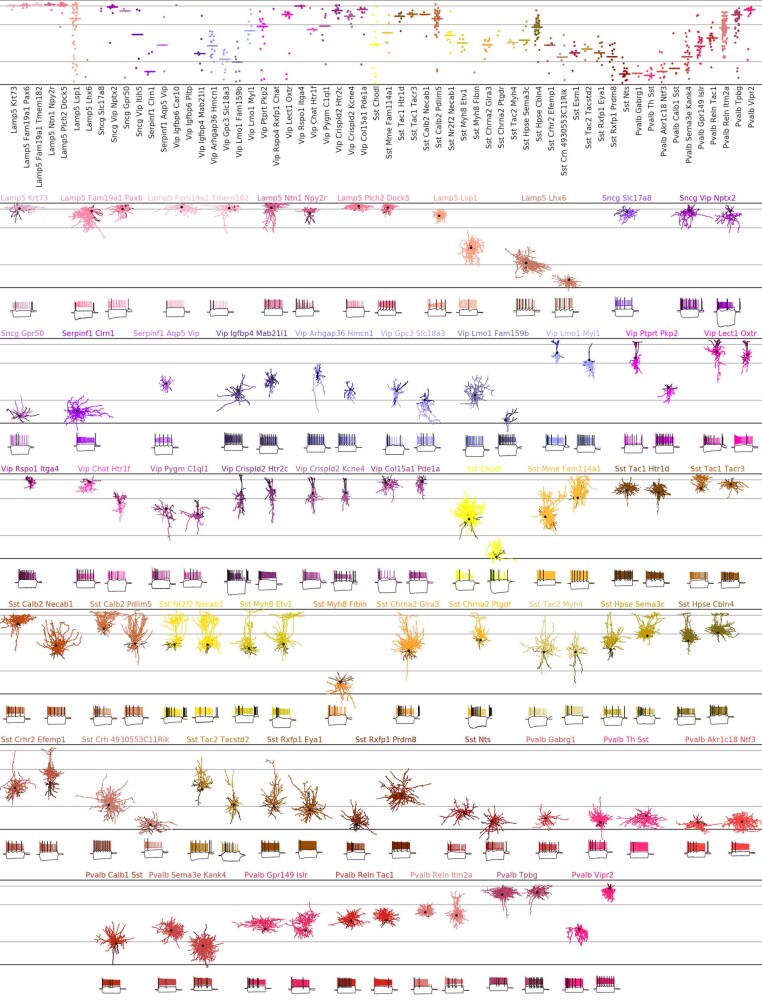
Interneurons assigned to the Tasic et al.^4^ t-types. This is an exact analogue of Fig. 1b and Extended Data Fig. 3 using inhibitory t-types from ref. ^4^. It allows the direct comparison with the results from ref. ^45^. We used the same neurons as in Extended Data Fig. 3 whenever possible. 99 neurons in 55 t-types.

A parallel Patch-seq study of the inhibitory neurons in the mouse visual cortex^45^ focused on isolating multimodal neural types (‘met-types’) but also often observed continuous variation. Our data sets are overall in good agreement (Extended Data Fig. 9) and together offer an unprecedented view of cell type variability in the neocortex. Future studies will need to add additional modalities, such as long-range projections, local connectivity, and in vivo functional characterization.

## Methods

No statistical methods were used to predetermine sample size. The experiments were not randomized and investigators were not blinded to allocation during experiments and outcome assessment, unless otherwise stated.

### Animals

Experiments on adult male and female mice (*n* = 266; median age 75 days, interquartile range 64–100, full range 35–245 days, Extended Data Fig. 2a) were performed on wild-type C57Bl/6 (*n* = 27), *Viaat*-Cre/Ai9 (vesicular inhibitory amino acid transporter, encoded by the *Slc32a1* gene, *n* = 24), *Sst*-Cre/Ai9 (somatostatin, *n* = 75), *Vip*-Cre/Ai9 (vasoactive intestinal polypeptide, *n* = 46), *Pvalb*-Cre/Ai9 (parvalbumin, *n* = 76), *Npy*-Cre/Ai9 (neuropeptide Y, *n* = 2), *Vipr2*-Cre/Ai9 (vasoactive intestinal peptide receptor 2, *n* = 7), *Scl17a8*-Cre/Ai9 (VGLUT3, vesicular glutamate transporter 3, *n* = 6), *Gnb4*-Cre/Ai9 (*n* = 1), and *Slc17a8*-iCre/Ai9 (*n* = 2) mice. Numbers above refer to mice from which sequencing data were successfully obtained. Several more animals were used for measuring layer boundaries and follow-up experiments at physiological temperature (see below). Mice were co-housed with littermates (2–5 per cage) in a controlled environment at 22–24 °C and 30–70% humidity. Mice were maintained with unrestricted access to food and water on a 12-h light/dark cycle. Procedures for mouse maintenance and surgeries were performed according to protocols approved by the Institutional Animal Care and Use Committee (IACUC) of Baylor College of Medicine.

The *Viaat*-Cre line was generously donated by Huda Zoghbi (Baylor College of Medicine), the *Slc17a8*-iCre line by Rebecca Seal (University of Pittsburg). The *Gnb4*-Cre line was  from the Allen Institute for Brain Science. The other Cre and reporter lines were purchased from the Jackson Laboratory: *Sst*-Cre (stock no. 013044), *Vip*-Cre (stock no. 010908), *Pvalb*-Cre (stock no. 008069), *Vipr2*-Cre (stock no. 031332), *Slc17a8*-Cre (stock no. 028534), *Npy*-Cre (stock no. 027851), Ai9 reporter (stock no. 007909).

We were unable to find any labelled cells in MOp in the *Gnb4*-Cre mice: all labelled cells were far outside of MOp and close to the claustrum^46^. For this reason, the data set does not include any *Gnb4*-positive cells.

### Slice preparation

The MOp brain slices were obtained following previously described protocols^5,28^. In brief, the animals were deeply anaesthetized using 3% isoflurane and decapitated. The brain was rapidly removed and collected into cold (0–4 °C) oxygenated NMDG (*N*-methyl-d-glucamine) solution containing 93 mM NMDG, 93 mM HCl, 2.5 mM KCl, 1.2 mM NaH_2_PO_4_, 30 mM NaHCO_3_, 20 mM HEPES, 25 mM glucose, 5 mM sodium ascorbate, 2 mM thiourea, 3 mM sodium pyruvate, 10 mM MgSO_4_ and 0.5 mM CaCl_2_, pH 7.35 (all from Sigma-Aldrich). We cut 300-μm-thick coronal slices using a Leica VT1200 microtome following coordinates provided in the Allen Brain Atlas for adult mouse (http://atlas.brain-map.org). The slices were subsequently incubated at 34.0 ± 0.5 °C in oxygenated NMDG solution for 10–15 min before being transferred to the artificial cerebrospinal fluid (ACSF) solution containing: 125 mM NaCl, 2.5 mM KCl, 1.25 mM NaH_2_PO_4_, 25 mM NaHCO_3_, 1 mM MgCl_2_, 11.1 mM glucose and 2 mM CaCl_2_, pH 7.4 (all from Sigma-Aldrich) for about 1 h. The slices were allowed to recover in ACSF equilibrated with CO_2_/O_2_ gas mixture (5% CO_2_, 95% O_2_), at room temperature (approximately 25 °C) for 1 h before experiments. During the recordings, slices were submerged in a customized chamber continuously perfused with oxygenated physiological solution. Recorded cells were generally located 15–60 μm deep under the slice surface.

### Patch-seq recording procedures

In order to simultaneously obtain electrophysiological, morphological and transcriptomic data from the same neurons, we applied our recently developed Patch-seq protocol^17^, with some modifications. In particular, changes were made to the internal solution to optimize its osmolarity in order to improve staining quality. RNase-free intracellular solution was prepared as follows: we dissolved 111 mM potassium gluconate, 4 mM KCl, 10 mM HEPES and 0.2 mM EGTA in RNase-free water in a 125-ml Erlenmeyer flask. We then covered the solution with aluminium foil and autoclaved it. After the solution was cooled down to room temperature, we added 4 mM MgATP, 0.3 mM Na_3_GTP, 5 mM sodium phosphocreatine, and 13.4 mM biocytin (all from Sigma-Aldrich). The pH was adjusted to 7.25 with RNase-free 0.5 M KOH using a dedicated pH meter (cleaned with RNase Zap and RNase-free water before each use). RNase-free water was then added to the solution in order to obtain the desired volume. After carefully checking its osmolarity (approximately 235–240 mOSM) the solution was stored at −20 °C and used for no longer than 3 weeks.

Before each experiment, we combined 494 μl internal solution with 6 μl recombinant RNase inhibitor (1 U/μl, Takara) to increase RNA yield. The addition of the inhibitor resulted in an increase in osmolarity to the desired value of 315–320 mOSM without a further dilution^17^. The osmolarity of the ACSF was monitored before each experiment and adjusted to be 18–20 mOSM lower than the internal solution. In particular, when the ACSF osmolarity was too low, we added a small amount of sucrose to ACSF to increase its osmolarity and bring it to the desired range. This osmolarity difference between ACSF and the internal solution is important to obtain slight swelling of the cell during the recording session, which improves the diffusion of biocytin in the neuronal processes. All glassware, spatulas, stir bars, counters, and anything else that may come into contact with the reagents or solution were cleaned thoroughly with RNase Zap before use.

Recording pipettes (B200-116-10; Sutter Instrument) of ~3–7 MΩ resistance were filled with 0.1–0.3 μl RNase-free intracellular solution. The size of the pipette tip was chosen according to the target neuron size: 3–4-MΩ pipettes were used to record large neurons (for example, L5 ET excitatory neurons) and 6–7-MΩ pipettes were used to record small cells such as L1 or *Vip* interneurons.

The PatchMaster software (HEKA Elektronik) and custom Matlab scripts were used to operate the Quadro EPC 10 amplifiers and to perform online and offline data analysis. We used the following quality control criteria: (1) seal resistance value >1 GΩ before achieving whole-cell configuration; (2) access resistance <30 MΩ. Each neuron was injected with 600-ms-long current pulses starting from −200 pA and up to 1,380 pA with 20-pA increment steps (in some cases stimulation was stopped before reaching 1,380 pA). There were 1.3- or 1.4-s intervals between successive current pulses, depending on the used setup. For most neurons, the stimulation was then repeated multiple times from the beginning. Electrophysiological traces used for the analysis were acquired between 3 and 15 min after achieving the whole-cell configuration. Recordings were performed at room temperature (25 °C), as opposed to physiological temperature (34 °C), in order to keep the cells alive for longer. We performed control experiments at physiological temperature as well (see below).

Typically, excitatory neurons were recorded for 5–20 min while interneurons were recorded for 20–50 min in order to allow biocytin to diffuse into distal axonal segments. During the recording, the access resistance was checked every three minutes in order to maintain a stable seal that would ensure successful biocytin diffusion. The resulting cDNA yield was not correlated with the hold time (Spearman correlation −0.01).

### Experiments at physiological temperature

A subset of electrophysiological recordings was performed at 34 °C in the presence of fast glutamatergic and GABAergic synaptic transmission blockers, 1 mM kynurenic acid (Sigma-Aldrich) and 0.1 mM picrotoxin (Tocris), respectively. The temperature was maintained stable, and constantly monitored using the temperature controller TC07 (Luigs and Neumann). In this set of experiments, the morphologies were not recovered and multiple neurons were recorded in each slice. The soma depth and the slice thickness were measured before each recording using Linlab2 software (Scientifica). Intrinsic electrophysiological recordings were obtained using the same stimulation paradigm as described above.

In these experiments, we targeted L5 *Sst* and excitatory neurons (Extended Data Fig. 6). We sequenced in total 185 neurons, obtained from 8 adult mice (7 *Sst*-Cre/Ai9 and 1 *Pvalb*-Cre/Ai9), of which 177 neurons passed the transcriptomic quality control and got a t-type assignment (see below). One hundred and ten cells mapped to the *Sst* subclass, 43 to IT, 12 to ET, 10 to *Pvalb*, and 2 to NP. 175 cells were assigned to L5 in the post hoc analysis (see below). We obtained high-quality electrophysiological recordings and extracted electrophysiological features of 184 cells.

### RNA sequencing of patched cells

At the end of the recording session, cell contents were aspirated into the glass pipette by applying a gentle negative pressure (0.7–1.5 pounds per square inch) for 1–5 min until the size of the cell body was visibly reduced. In most cases, the cell nucleus was visibly attached to the pipette tip and extracted from the cell body. We avoided complete nucleus aspiration, because it can lead to the collapse of the soma structure and of the nearby neurites, resulting in lower staining quality and stronger background staining. During the aspiration process, the cell body structure and access resistance were constantly monitored. Special care was taken to ensure that the seal between the pipette and the cell membrane remained intact to reduce contamination from the extracellular environment. After aspiration, the contents of the pipette were immediately ejected into a 0.2-ml PCR tube containing 4 μl lysis buffer (with ERCC spike-ins), and RNA was subsequently converted into cDNA using a Smart-seq2-based protocol^21^ as described previously^17^. The resulting cDNA libraries were screened using an Agilent Bioanalyzer 2100. Samples containing less than around 1 ng total cDNA (in the 15 μl final volume) or with an average size less than 1,500 bp were typically not sequenced (with some occasional exceptions). The cDNA libraries were then frozen and sent for sequencing in 12 separate batches.

The cDNA libraries derived from each neuron were purified and 0.2 ng of the purified cDNA was tagmented using the Illumina Nextera XT Library Preparation with one-fifth of the volumes stated in the manufacturer’s recommendation. Custom 8-bp index primers were used at a final concentration of 0.1 μM. The resulting cDNA library of each batch was sequenced on an Illumina NextSeq500 instrument with a sequencing setup of 75-bp single-end reads and 8-bp index reads. The investigators were blinded to the cell type of each sample during library construction and sequencing.

The sequencing data were processed using the zUMIs 2.5.6b pipeline with default settings^47^. Sequencing reads were aligned to the mm10 mouse reference genome using STAR version 2.5.4b^48^ and transcript assignment performed with Gencode transcript annotations, version M23. A substantial portion of the RNA extracted from the neurons was nascent and contained intronic sequences. To accommodate this, gene expression counts were separately calculated using reads mapping to annotated intronic and exonic regions. We detected 42,466 genes, including pseudogenes and annotated non-coding segments, in at least one cell. The resulting exonic and intronic read count data were used for all transcriptomic analyses presented here. To quantify gene expression, we typically normalized exon and intron counts by exonic and intronic gene lengths in kilobases and added normalized counts together to obtain normalized exonic + intronic expression levels. See below for more details. Throughout the manuscript, ‘detected gene’ refers to a gene with a non-zero exonic or intronic count.

### Biocytin staining and morphological reconstructions

Morphological recovery was carried out as previously described^5,17,28^. In brief, after the recordings, the slices were immersed in freshly prepared 2.5% glutaraldehyde, 4% paraformaldehyde solution in 0.1 M PBS at 4 °C for at least 48 h. The slices were subsequently processed with the avidin-biotin-peroxidase method to reveal the morphology of the neurons. As previously described, we took several steps to improve the staining quality of the fine axonal branches of interneurons^5,17^. First, we used a high biocytin concentration (0.5 g/100 ml). Second, we incubated with avidin–biotin complex and detergents at a high concentration (Triton X-100, 5%) for at least 24 h before staining with 3,3′-diaminobenzidine (DAB).

Recovered cells were manually reconstructed using a 100 × oil-immersion lens and a camera lucida system (MicroBrightField). We aimed to reconstruct all cells that had staining of sufficient quality (axons and dendrites for the inhibitory neurons; only dendrites for the excitatory neurons), and obtained 646 reconstructions in total. In addition, we reconstructed the dendrites of 30 neurons from the *Vip* and *Scng* subclasses that lacked sufficient axonal staining. *Vip* neurons are traditionally classified on the basis of dendritic morphology, so these reconstructions can inform t-type characterizations. These additional 30 reconstructions are shown, together with the main 646 reconstructions, in Supplementary File 1.

Forty-five sequenced cells were mistakenly recorded using a solution with a much smaller concentration of biocytin, and their morphologies could not be recovered. We made sure that the measured electrophysiological properties of these cells were not systematically different from those of the the other sequenced cells.

Inevitably, neuronal structures can be severed as a result of the slicing procedure. We took special care to exclude reconstructions of all neurons that showed any signs of damage, lack of contrast, or poor overall staining. Consistently with previous studies, tissue shrinkage due to the fixation and staining procedures was about 10–20%^5,28,49^. This shrinkage was not compensated for in our analysis.

### Cortical thickness normalization and layer assignment

Nissl-stained slices (*n* = 15 from two wild-type adult mice) were used to measure normalized layer boundaries in MOp. The Nissl staining protocol was adapted from ref. ^50^. In brief, brain slices were mounted on slides and allowed to dry. The sections were then demyelinated, stained with 0.1% cresyl violet-acetate (C5042, Sigma) for 30 min at 60 °C and further destained. The sections were then coverslipped in Cytoseal 60 (Richard Allan Scientific). For each slice we measured total thickness from pia to white matter and the depths of the three between-layer boundaries (L1 to L2/3, L2/3 to L5, L5 to L6), based on the cortical cytoarchitecture, using a Neurolucida system with 10 × or 20 × magnification. All measurements were normalized by the respective slice thickness, and the averages over all *n* = 15 slices were used as the normalized layer boundaries (Extended Data Fig. 2b).

For the Patch-seq neurons, we measured soma depth and the cortical thickness of the slice using a Neurolucida system. We took their ratio as the normalized soma depth, and assigned each neuron to a layer (L1, L2/3, L5, or L6) based on the Nissl-determined layer boundaries (Extended Data Fig. 2b). We obtained soma depth information for 1,284 neurons out of 1,329 (45 neurons were mistakenly recorded using a solution with insufficient biocytin concentration, and we could measure soma depths for only 2 of those; for 2 other neurons the measurements could not be carried out because the slices were lost). For the 45 neurons with missing soma depth measurements, we used the layer targeted during the recording for all layer-based analyses and visualizations (marker shapes in Figs. 1c–e, 3a–c, layer-restricted analysis in Fig. 4, Extended Data Fig. 8).

All reconstructed morphologies were normalized by the cortical thickness of the respective slice to make it possible to display several morphologies next to each other, as in Extended Data Fig. 3.

### t-Type assignment

The t-type assignment procedure was done in two rounds. The first round was for quality control and initial assignment to one of the three large transcriptomic groups (CGE-derived interneurons, MGE-derived interneurons, and excitatory neurons) that are perfectly separated from each other with no transcriptomically intermediate cells^4^. The second round was done to assign the cells to specific t-types.

In the first round, we mapped each Patch-seq cell to a large annotated Smart-seq2 reference data set from adult mouse cortex^4^, using a procedure similar to the one described in ref. ^28^. Specifically, using the exon count matrix of the reference data set, we selected the 3,000 most variable genes (see below). We then normalized all exon counts by exonic gene lengths in kilobases, all intron counts by intronic gene lengths in kilobases (plus 10^−6^, to avoid division by zero) and added normalized counts together to obtain normalized exonic + intronic expression levels. We log-transformed these values using log_2_(*x* + 1) transformation and averaged the log-transformed values across all cells in each of the 133 t-types, to obtain reference transcriptomic profiles of each t-type (133 × 3,000 matrix). Out of these 3,000 genes, 2,666 were present in the genome annotation that we used and were detected in our data set. We applied the same normalization and log-transformation procedure to the exonic and intronic read counts of our cells, and for each cell computed Pearson correlation across the 2,666 genes with each of the 133 t-types. Each cell was assigned to the t-type to which it had the highest correlation (Extended Data Fig. 1d).

Cells meeting any of the following exclusion criteria were declared low quality and did not get a t-type assignment (Extended Data Fig. 2e): cells with the highest correlation below 0.4 (78 cells); cells that would be assigned to non-neural t-types, presumably owing to RNA contamination^51^ (14 cells; see also Extended Data Fig. 2j–n); cells with the highest correlation less than 0.02 above the maximal correlation in one of the other two large transcriptomic groups (5 cells). The remaining 1,232 cells passed quality control and entered the second round.

In the second round, cells were independently mapped to the seven transcriptomic data sets obtained from mouse MOp^20^. The mapping was done only to the t-types from the transcriptomic group identified in the first round, using the 500 most variable genes in that data set for that transcriptomic group (so using 7 × 3 = 21 sets of 500 most variable genes). Gene selection was performed as described below, and t-type assignment was done exactly as described above. Across the 21 reference subsets, 421–494 most variable genes were present in our data set, and were used for the t-type assignment (Extended Data Fig. 1e). When mapping to the Smart-seq2 reference data sets, we used normalized intronic and exonic reference counts, as above. When mapping to the UMI-based reference data sets, we used the unique molecular identifier (UMI) counts directly, without gene length normalization.

We used bootstrapping over genes to assess the confidence of each t-type assignment. For each cell and for each of the seven reference data sets, we repeatedly selected a bootstrap sample of genes (that is, the same number of genes, selected randomly with repetitions) and repeated the mapping. This was done 100 times and the fraction of times the cell mapped to each t-type was taken as the t-type assignment confidence for that t-type (Extended Data Fig. 1f). The confidences obtained with seven reference data sets agreed well with each other (Extended Data Fig. 2i) and were averaged to obtain the consensus confidence. Finally, the cell was assigned to the t-type with the highest consensus confidence.

Four cells were assigned to an excitatory t-type, despite having clearly inhibitory firing, morphology, and/or soma depth location (such as L1). The most likely cause of this was RNA contamination from excitatory cells, which are much more abundant than inhibitory cells in the mouse cortex (Extended Data Fig. 2). These four cells were excluded from all analyses and visualizations (as if they did not pass the transcriptomic quality control). In addition, one cell was probably located outside MOp, based on the slice anatomy, and was excluded as well. The final number of cells with t-type assignment was 1,227.

### Selection of most variable genes

Several steps of our analysis required selecting a set of the most variable genes in a given transcriptomic data set. We always selected a fixed predefined number of genes (such as 500, 1,000, or 3,000).

To select the most variable genes, we found genes that had, at the same time, high non-zero expression and a high probability of near-zero expression^52^. Our procedure is described in more detail elsewhere^23^. Specifically, we excluded all genes that had counts of at least *c*_min_ (for Patch-seq and Smart-seq2: *c*_min_ = 32; for 10x: *c*_min_ = 0) in fewer than 10 cells. For each remaining gene we computed the mean log_2_ count across all counts that were larger than *c*_min_ (non-zero expression, *μ*) and the fraction of counts that were smaller than or equal to *c*_min_ (probability of near-zero expression, *τ*). Across genes, there was a clear inverse relationship between *μ* and *τ*, that roughly followed the exponential law: *τ* ≈ exp(−1.5 × *μ* + *a*) for some horizontal offset *a*. Using a binary search, we found a value *b* of this offset that yielded the desired number of genes with *τ* > exp(−1.5 × *μ* + *b*) + 0.002.

For Smart-seq2 and Patch-seq data sets, we used only exonic counts to perform gene selection.

### t-SNE visualization of the transcriptomic data

t-SNE embeddings^22^ of the three subsets of the single-cell 10x v2 data set^20^ (Fig. 1c–e) were constructed using the same 500 most variable genes that were used for t-type assignment (see above). The UMI counts were normalized by each cell’s sequencing depth (sum of counts), multiplied by the median sequencing depth across all cells, log_2_(*x* + 1)-transformed, and reduced to 50 principal components. The resulting *n* × 50 matrix was used as input to t-SNE. We used FIt-SNE 1.2.1^53^ with default parameters (including learning rate *n*/12 and scaled principal component analysis (PCA) initialization^23^). Perplexity was left at the default value of 30 for both inhibitory subsets and increased to 100 for the excitatory subset.

To position Patch-seq cells on a reference t-SNE embedding, we used a published procedure^23^. In brief, each cell was positioned at the median embedding location of its ten nearest neighbours, based on Pearson correlation distance in the high-dimensional space. As above, we used the sum of the normalized exonic and intronic counts for Patch-seq cells, and raw UMI counts for the reference cells. All values were log_2_(*x* + 1)-transformed and correlations were computed across the same genes that were used for t-type assignments (see above).

### Extraction of electrophysiological features

Twenty-nine electrophysiological properties of the neurons were automatically extracted based on the raw membrane voltage traces (Extended Data Fig. 4) using Python scripts from the Allen Software Development Kit (SDK) (https://github.com/AllenInstitute/AllenSDK) with some modifications to account for our experimental paradigm (https://github.com/berenslab/EphysExtraction).

For each hyperpolarizing current injection, the resting membrane potential was computed as the mean membrane voltage during 100 ms before stimulation onset and the input resistance as the difference between the steady state voltage and the resting membrane potential, divided by the injected current value (we took the average voltage of the last 100 ms before stimulus offset as steady state). The median of these values over all hyperpolarizing traces was taken as the final resting membrane potential and input resistance, respectively.

To estimate the rheobase (the minimum current needed to elicit any spikes), we used robust regression (random sample consensus algorithm, as implemented in sklearn.linear_model.RANSACRegressor) of the spiking frequency onto the injected current using the five lowest depolarizing currents with non-zero spike count (if there were fewer than five, we used those available). The point at which the regression line crossed the *x*-axis gave the rheobase estimate (Extended Data Fig. 4). We restricted it to be between the highest injected current that elicited no spikes and the lowest injected current that elicited at least one spike. If the regression line crossed the *x*-axis outside this interval, the first current step that elicited at least one spike was used.

The action potential (AP) threshold, AP amplitude, AP width, afterhyperpolarization (AHP), afterdepolarization (ADP), the first AP latency, and the upstroke-to-downstroke ratio (UDR) were computed as illustrated in Extended Data Fig. 4, using the first AP fired by the neuron. AP width was computed at the AP half-height. UDR refers to the ratio of the maximal membrane voltage derivative during the AP upstroke to the maximal absolute value of the membrane voltage derivative during the AP downstroke. We also computed the first AP latency at 20 pA current above the smallest current stimulation value that elicited a spike.

The interspike interval (ISI) adaptation index for each trace was defined as the ratio of the second ISI to the first one. The ISI average adaptation index was defined as the mean of ISI ratios corresponding to all consecutive pairs of ISIs in that trace. For both quantities we took the median over the five lowest depolarizing currents that elicited at least three spikes (if fewer than five were available, we used all of them). AP amplitude adaptation index and AP amplitude average adaptation index were defined analogously to the two ISI adaptation indices, but using the ratios of consecutive AP amplitudes (and using the median over the five lowest depolarizing currents that elicited at least two spikes).

The maximum number of APs refers to the number of APs emitted during the 600-ms stimulation window of the highest firing trace. The spike frequency adaptation (SFA) denotes the ratio of the number of APs in the second half of the stimulation window to the number of APs in the first half of the stimulation window of the highest firing trace. If the highest firing trace had fewer than five APs, SFA was not defined. Here and below the highest firing trace corresponds to the first depolarizing current step that showed the maximum number of APs during the current stimulation window (after excluding all stimulation currents for which at least one AP was observed in 100 ms before or in 200 ms after the stimulation window; see below).

The membrane time constant (*τ*) was computed as the time constant of the exponential fit to the membrane voltage from the stimulation onset to the first local minimum (we took the median over all hyperpolarizing traces). Three further features described the sag of the first (the lowest) hyperpolarization trace. The sag ratio was defined as the difference between the sag trough voltage (average voltage in a 5-ms window around the sag trough) and the resting membrane potential, divided by the steady state membrane voltage difference from the resting membrane potential. The sag time was defined as the time period between the first and the second moments at which the membrane voltage crossed the steady-state value after the stimulation onset. The sag area refers to the absolute value of the integral of the membrane voltage minus the steady-state voltage during the sag time period (Extended Data Fig. 4). If the sag trough voltage and the steady-state voltage differed by less than 4 mV, the sag time and sag area were set to zero.

The rebound was defined as the voltage difference between the resting membrane potential and the average voltage over 150 ms (or whatever time remained until 300 ms after the stimulation offset) after rebound onset, which we identified as the time point after stimulation offset at which the membrane voltage reached the value of the resting membrane potential. If the membrane voltage never reached the resting membrane potential during the 300 ms after the stimulation offset, the rebound was set to zero. The rebound number of APs was defined as the number of APs emitted during the same period of time. Both rebound features were computed using the lowest hyperpolarization trace.

The ISI coefficient of variation (CV) refers to the standard deviation divided by the mean of all ISIs in the highest firing trace. Note that a Poisson firing neuron would have ISI CV equal to one. The ISI Fano factor refers to the variance divided by the mean of all ISIs in the highest firing rate. The AP CV and AP Fano factor refer to the CV and the Fano factor of the AP amplitudes in the highest firing trace, respectively.

The burstiness was defined as the difference between the inverse of the smallest ISI within a detected burst and the inverse of the smallest ISI outside bursts, divided by their sum. We took the median over the first five depolarizing traces. We relied on the Allen SDK code to detect the bursts. In brief, within that code a burst onset was identified whenever a ‘detour’ ISI was followed by a ‘direct’ ISI. Detour ISIs are ISIs with a non-zero ADP or a drop of at least 0.5 mV of the membrane voltage after the first AP terminates and before the next one is elicited. Direct ISIs are ISIs with no ADP and no such drop of membrane voltage before the second AP. A burst offset was identified whenever a direct ISI was followed by a detour ISI. Additionally, bursts were required to contain no ‘pauselike’ ISIs, defined as unusually long ISIs for that trace (see Allen SDK for the implementation details).

Some neurons (in particular neurogliaform cells) started to emit APs before and after the current stimulation window, after the stimulation currents exceeded a certain amount. To quantify this effect, we defined wildness as the difference in the number of APs between the highest firing trace (possibly showing APs before or after the stimulation window) and the highest firing trace as defined above (without any APs outside the stimulation window). For most neurons, wildness was equal to zero.

For all statistical analysis we used 17 features out of the extracted 29, excluding features that were equal to zero for many cells (afterdepolarization, burstiness, rebound number of APs, sag area, sag time, wildness), two Fano factor features that were highly correlated with the corresponding coefficient of variation features (AP Fano factor, ISI Fano factor) and another measure of latency that was highly correlated with the latency itself, features that had very skewed distributions (AP amplitude average adaptation index, ISI average adaptation index), and features that were undefined for some of the cells (spike frequency adaptation). Four features were log-transformed to make their distribution more Gaussian-like: AP coefficient of variation, ISI coefficient of variation, ISI adaptation index, and latency.

### Extraction of morphological features

Reconstructed morphologies were converted into the SWC format using NLMorphologyConverter 0.9.0 (http://neuronland.org) and further analysed using MorphoPy (https://github.com/berenslab/MorphoPy, version 0.6)^54^. Each cell was soma-centred in the *x* (slice width) and *y* (slice depth) dimensions, and aligned to pia in the *z* (cortical depth) dimension so that *z* = 0 corresponded to pia. All neurites were smoothed in the slice depth dimension (*y*) using a Savitzky–Golay filter of order 3 and window length 21, after resampling points to have maximally 1 μm spacing. For further analysis we computed two different feature representations of each cell: the normalized *z*-profile and a set of morphometric statistics^24,28,55^.

To compute the normalized *z*-profile, we divided all the coordinates of the neuronal point cloud by the thickness of the respective cortical slice, so that *z* = 1 corresponded to the white matter border. We projected this point cloud onto the *z*-axis and binned it into 20 equal-sized bins spanning [0, 1]. The resulting histogram describes a neuron’s normalized depth profile perpendicular to the pia. For the purposes of downstream analysis, we treated this as a set of 20 features. The *z*-profiles were separately computed for axons and dendrites.

Morphometric statistics were separately computed for the dendritic and axonal neurites to quantify their arborization shape and branching patterns. For the excitatory neurons, several additional morphometric statistics were computed for the apical dendrites, where apical dendrite was operationally defined as the dendrite with the longest total path length. We further used two ‘somatic’ features: normalized soma depth and soma radius. We did not use any features measuring morphological properties in the slice depth (*y*) direction because of possible slice shrinkage artefacts. We did not use any axonal features for the excitatory cells because only a small part of the axon could typically be reconstructed. For the inhibitory cells, where dendrite and axon could both be fully recovered, we included some measures of dendritic and axonal overlap. The full list of morphometric statistics is given in Supplementary File 3.

We extracted a set of 75 features, of which 40 were defined for excitatory neurons and 62 for inhibitory neurons, and processed the data for excitatory and inhibitory neurons separately. In each case, we excluded features with coefficient of variation below 0.25 (among the features with only positive values). This procedure excluded five features for the excitatory and nine features for the inhibitory cells. The distributions of the remaining features were visually checked for outliers and for meaningful variation between transcriptomic types, leading to a further exclusion of three features for the inhibitory cells. The full list of excluded features is given in Supplementary File 3. The resulting set of morphometric statistics used for further analysis consisted of 35 features defined for the excitatory neurons and 50 features defined for the inhibitory neurons.

### Reduced-rank regression

For the RRR analysis^32^ we used 17 electrophyiological features and all 1,219 cells for which values for all 17 features and a t-type assignment could be computed. Electrophysiological features were standardized. Exon counts and intron counts were normalized by the exon/intron gene lengths as described above, summed together, converted to CPM, log_2_(*x* + 1)-transformed, and then standardized. We selected the 1,000 most variable genes (using raw exonic counts) and used only those for the RRR analysis.

In brief, RRR finds a linear mapping of gene expression levels to a low-dimensional latent representation, from which the electrophysiological features are then predicted with another linear transformation (for mathematical details, see ref. ^32^). The model uses sparsity constraints in the form of elastic net penalty to select only a small number of genes. For Fig. 2 we used a model with rank *r* = 5, zero ridge penalty (*α* = 1), and lasso penalty tuned to yield a selection of 25 genes (*λ* = 0.5). Cross-validation (Extended Data Fig. 5) was done using 10 folds, elastic net *α*-values 0.5, 0.75, and 1.0, and *λ*-values from 0.2 to 6.0.

The plots shown in Fig. 2a, b are called bibiplots because they combine two biplots: the left biplot shows a mapping of gene expression levels onto the two latent dimensions; the right biplot shows the same mapping of electrophysiological features. To illustrate the meaning of the latent dimensions, each biplot combines the resulting scatter plots with lines showing how original features are related to the latent dimensions. Specifically, we computed the correlations of individual genes or electrophysiological properties with the latent dimensions and visualized these correlations as lines on the biplot. The circle shows the maximal possible correlation; only lines longer than 0.4 times the circle radius are shown in Fig. 2. Label positions were automatically adjusted by simulating repulsive forces between all overlapping pairs of labels, until there was no overlap.

For the model based on ion channel genes, we obtained the list of 328 ion channel genes from https://www.genenames.org/data/genegroup/#!/group/177and used all 307 of them that had non-zero expression in at least 10 of our cells. We used rank *r* = 5, *α* = 1, and *λ* tuned to yield 25 genes (*λ* = 0.303), as above.

### t-SNE visualization of the morpho-electric phenotypes

For the t-SNE visualization^22^ of the electrophysiological phenotypes, we used 17 features as described above and all *n* = 1,320 cells that had values for all 17 features. All features were standardized across this set of cells and transformed with PCA into a set of 17 PCs. We scaled the PCs by the standard deviation of PC1. We used the t-SNE implementation from scikit-learn Python library with the default perplexity (30), early exaggeration 4 (the default value 12 can be too large for small data sets), and scaled PCA initialization^23^. Fig. 3a shows *n* = 1,219 cells that had a t-type assignment.

For the t-SNE visualization of the morphological phenotypes, we combined morphometric statistics with the normalized *z*-profiles. The pre-processing, including PCA, was done separately for the excitatory and inhibitory neurons because they used different sets of morphometric statistics (see above). Only neurons with assigned t-types were used for this analysis. Two inhibitory neurons were left out because some of the morphometric statistics could not be extracted owing to insufficient dendritic recovery; this left 367 inhibitory neurons (with 50 morphometric features) and 269 excitatory neurons (with 35 morphometric features). All features were standardized and each set was reduced to 20 PCs. We scaled the PCs by the standard deviation of the respective PC1, to make the inhibitory and the excitatory PCs have comparable variances.

We used dendritic *z*-profiles for the excitatory neurons and axonal *z*-profiles for the inhibitory neurons. We reduced each set to five PCs, discarded PC1 (it was strongly correlated with the normalized soma depth and made the resulting embedding strongly influenced by the soma depth), and scaled the PCs by the standard deviation of the respective PC2. We stacked the 20 scaled morphometric PCs and the 4 scaled *z*-profile PCs to get a combined 24-dimensional representation, separately for the excitatory and for the inhibitory neurons. We then combined these representations into one block-diagonal 48-dimensional matrix. This procedure makes the excitatory and the inhibitory populations both have zero mean. To prevent overlap between these two populations, we added a small constant value of 0.25 to the excitatory block-diagonal block, leading to the strong excitatory–inhibitory separation in Fig. 3b. The t-SNE was performed exactly as described above.

For the t-SNE visualization of the morpho-electrical landscape, we stacked together the 48-dimensional morphological representation and the 16-dimensional electrophysiological representation obtained above, using only cells that had all morphological and all electrophysiologcal features (*n* = 628). We multiplied the electrophysiological block by √2 to put its total variance on a similar scale (it only consisted of one set of scaled PCs, whereas the morphological representation consisted of two sets of scaled PCs: morphometrics and *z*-profiles). The resulting 64-dimensional morpho-electrical representation was used for t-SNE, exactly as described above.

### kNN classification of transcriptomic families

To classify neurons into transcriptomic families on the basis of electrophysiological, morphological, or combined features (Figs. 3d, 5a, Extended Data Fig. 8a), we used a kNN classifier with *k* = 10 and Euclidean distance metric (taking the majority family among the *k* nearest neighbours). This is effectively a leave-one-out cross-validation procedure. For each data modality we took the exact same data representation that was used for computing t-SNE embeddings (Fig. 3a–c; see above). Note that the t-SNE algorithm is also based on nearest neighbours and makes all close neighbours attract each other in the embedding. We chose the kNN classifier as a simple but versatile non-parametric classifier that is directly related to the t-SNE embeddings. We did not use the *Sncg* and NP families owing to insufficient coverage in our data set (Fig. 1).

Fig. 3d shows the fraction of cells from each family that was classified into each family. Fig. 5a and Extended Data Fig. 8a show fractions of cells from each t-type that were classified into each family. For morphological and combined features, Extended Data Fig. 8a shows fractions of cells from the majority layer of each t-type. For example, the *Pvalb Reln* type occurred most often in L5, so only cells from that layer were taken for that type. Only t-types with at least ten cells (or at least ten layer-restricted cells) are shown.

### Within-family analysis

To study the relationship between transcriptomic and electrophyiological distances between pairs of t-types (Fig. 4c, d, Extended Data Figs. 6, 7), we took all t-types with five or more cells assigned to them (for Extended Data Fig. 7a: with ten or more). For each pair of t-types, transcriptomic distance was computed as the Pearson correlation between the average log_2_(*x* + 1)-transformed UMI counts in the single-cell 10x v2 data^20^. The 1,000 most variable genes across all neural types were used for Fig. 4c and Extended Data Fig. 7a, b, and the 500 most variable genes across the respective transcriptomic group (see above) were used for Fig. 4d and Extended Data Figs. 6i, j and 7c–n. Electrophysiological distance was computed as the Euclidean distance between the average feature vectors. Fig. 4d used the soma depth distance, computed as the absolute value of the difference between the average normalized soma depths.

### T-type variability analysis

The normalized total variance in Fig. 5b and Extended Data Fig. 8b was computed as follows. For each modality, we took the exact same data representation that was used for computing t-SNE embeddings (Fig. 3a–c; see above). For each t-type (or layer-restricted t-type; see above), we took the sum of its variances in all dimensions as the total variance and divided by the sum of variances in all dimensions across the whole data set:

$$\frac{{\sum }_{j}\frac{1}{|T|}{\sum }_{i\in T}{\left({X}_{ij}-\frac{1}{|T|}{\sum }_{i\in T}{X}_{ij}\right)}^{2}}{{\sum }_{j}\frac{1}{n}{\sum }_{i}{\left({X}_{ij}-\frac{1}{n}{\sum }_{i}{X}_{ij}\right)}^{2}},$$

where *X*_*ij*_ is a value of feature *j* of cell *i*, *n* is the total number of cells, and *T* is the set of cell numbers belonging to the given t-type. The value 0 indicates that all cells from this t-type have exactly identical features. The value 1 indicates that there is as much variance in this one t-type as in the whole data set. Only t-types with at least ten cells (or at least ten layer-restricted cells) are shown in Fig. 5b and Extended Data Fig. 8b.

To provide a sensible baseline for the range of possible normalized total variances in a population of morpho-electrically homogeneous types, we used a clustering analysis. For the cells of all the *K* t-types (or layer-restricted t-types) with at least ten cells in a given panel, we used the *k*-means algorithm to cluster them into *K* clusters, reasoning that these clusters should be as homogeneous as possible given the variability in our data set. We used the *k*-means implementation from scikit-learn with default parameters. We then computed the normalized total variance of each cluster as described above. Grey shading in Fig. 5b and Extended Data Fig. 8b shows the interval between the minimum and the maximum cluster variances. Note that the *k*-means algorithm directly minimizes within-cluster total variances.

We used the entropies of a Leiden clustering^35^ as an alternative way to approach the same question. For each modality, using the exact same data representation as above, we constructed its kNN graph with *k* = 10 and clustered it using the Leiden algorithm as implemented in the Python package leidenalg with RBConfigurationVertexPartition quality function and resolution parameter manually tuned to produce roughly the same number of clusters for each modality as in ref. ^24^. (Extended Data Fig. 8). For each t-type (or layer-restricted t-type), we then measured the entropy of the distribution of electrophysiological or morphological cluster IDs, after randomly subsampling the t-type to ten cells. Subsampling was done to eliminate a possible bias due to the t-type abundance. The whole procedure was repeated 100 times with different random seeds for the Leiden clustering and for the subsampling.

### Reporting summary

Further information on research design is available in the Nature Research Reporting Summary linked to this paper.

## Online content

Any methods, additional references, Nature Research reporting summaries, source data, extended data, supplementary information, acknowledgements, peer review information; details of author contributions and competing interests; and statements of data and code availability are available at 10.1038/s41586-020-2907-3.

### Supplementary information

Supplementary Information Supplementary File 1: All reconstructed morphologies and electrophysiological traces shown as in Extended Data Fig.3, sorted by transcriptomic type.

Reporting Summary

Supplementary Information Supplementary File 2: Dot plots of all electrophysiological features shown as in Extended Data Fig. 4.

Supplementary File 3 Supplementary File 3: Full list of computed morphometric statistics.

## Figures and Tables

**Extended Data Table 1:**
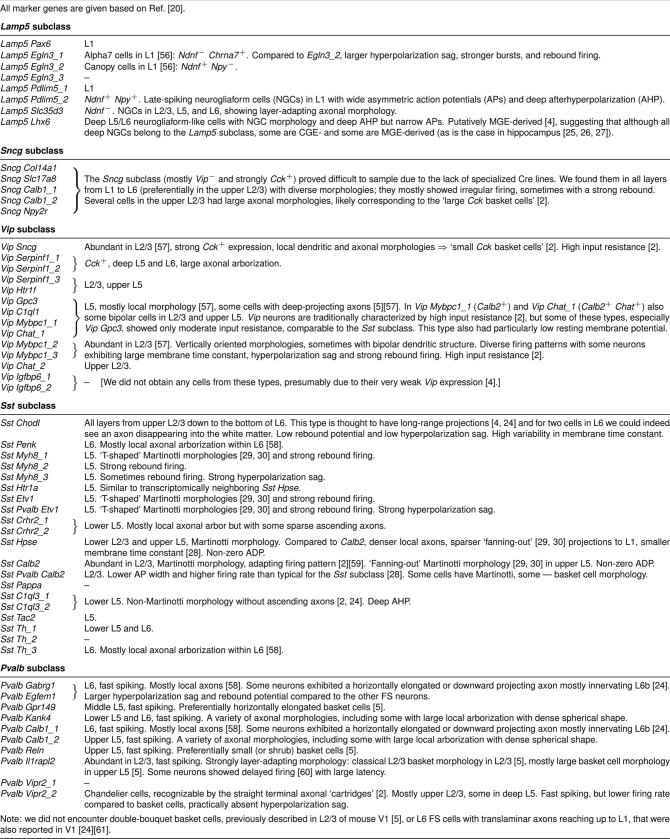
Description of the inhibitory t-types

References^56–61^ are cited in this table.

**Extended Data Table 2:**
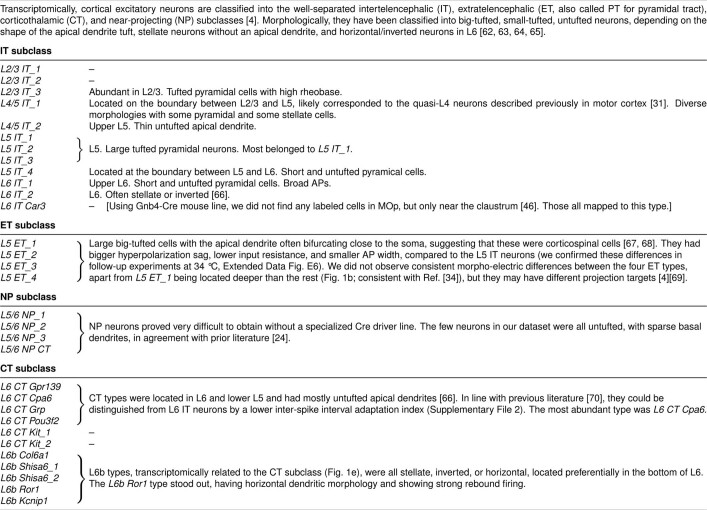
Description of the excitatory t-types

References^62–70^ are cited in this table.

## Data Availability

All preprocessed data (gene counts, electrophysiological and morphological features) and meta data are available at https://github.com/berenslab/mini-atlas, together with direct links to the raw data. Electrophysiological recordings are available at https://dandiarchive.org/dandiset/000008 (main data set) and https://dandiarchive.org/dandiset/000035 (physiological temperature) in NWB format. Sequencing data are available at http://data.nemoarchive.org/biccn/grant/zeng/tolias in FASTQ format. Morphological reconstructions are available at https://download.brainimagelibrary.org/3a/88/3a88a7687ab66069/ in SWC format.

## Code availability

The analysis code in Python is available at https://github.com/berenslab/mini-atlas.
