# Supplementary figures and images for: Phenotypic variation of transcriptomic cell types in mouse motor cortex
Source: Nature. 2020 Nov 12;598(7879):144–50. doi: 10.1038/s41586-020-2907-3 (PMC8113357; doi:10.1038/s41586-020-2907-3)

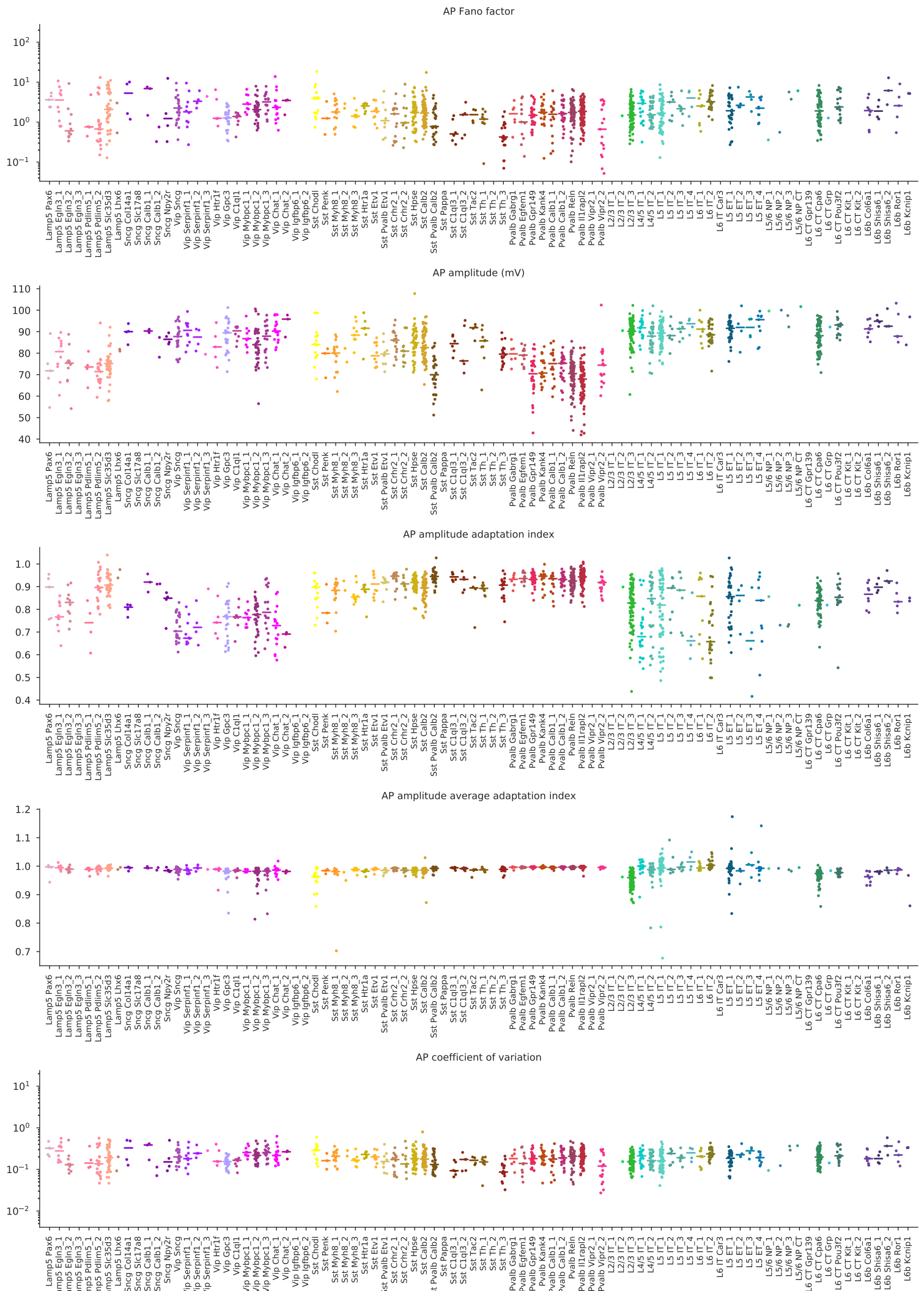

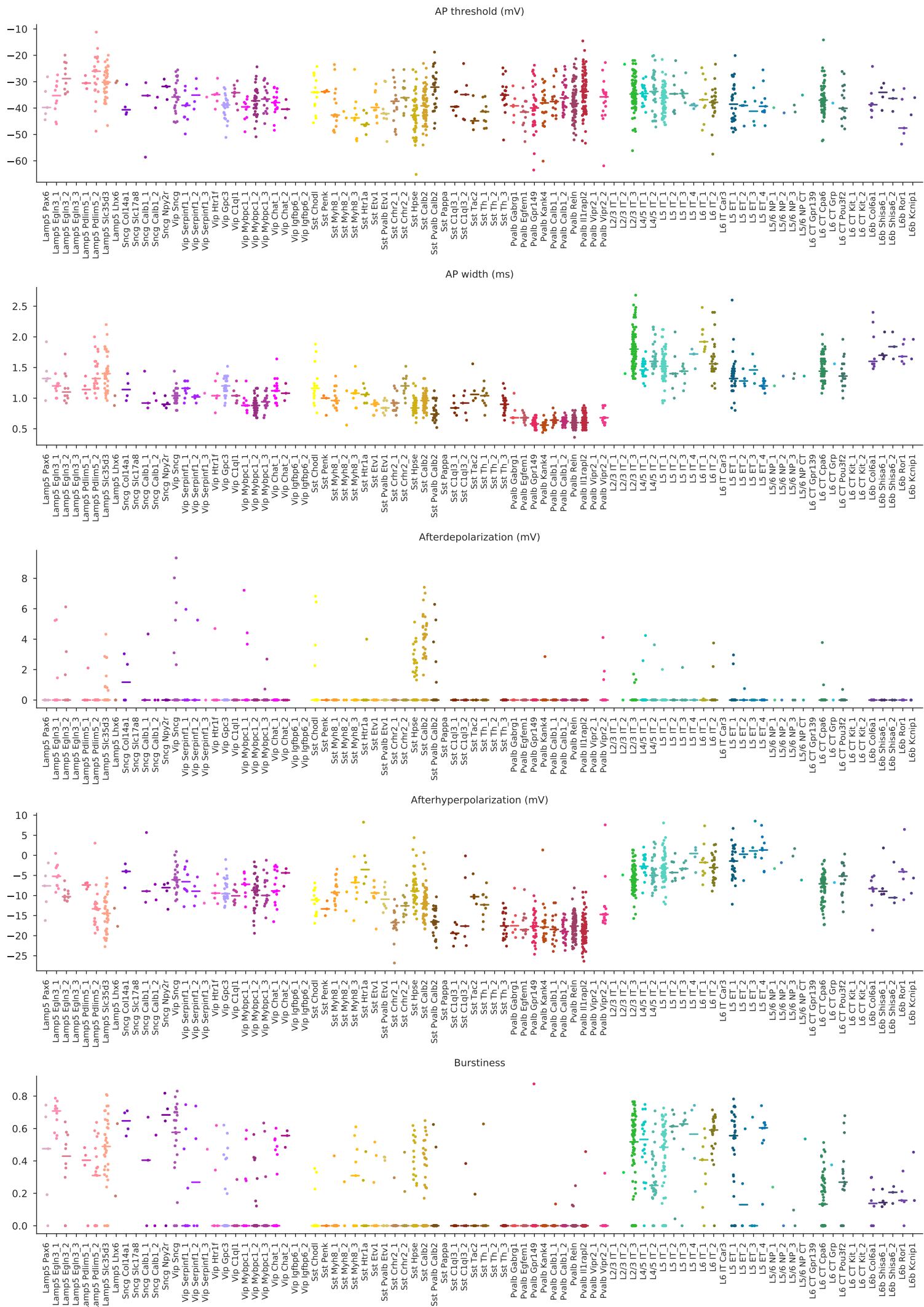



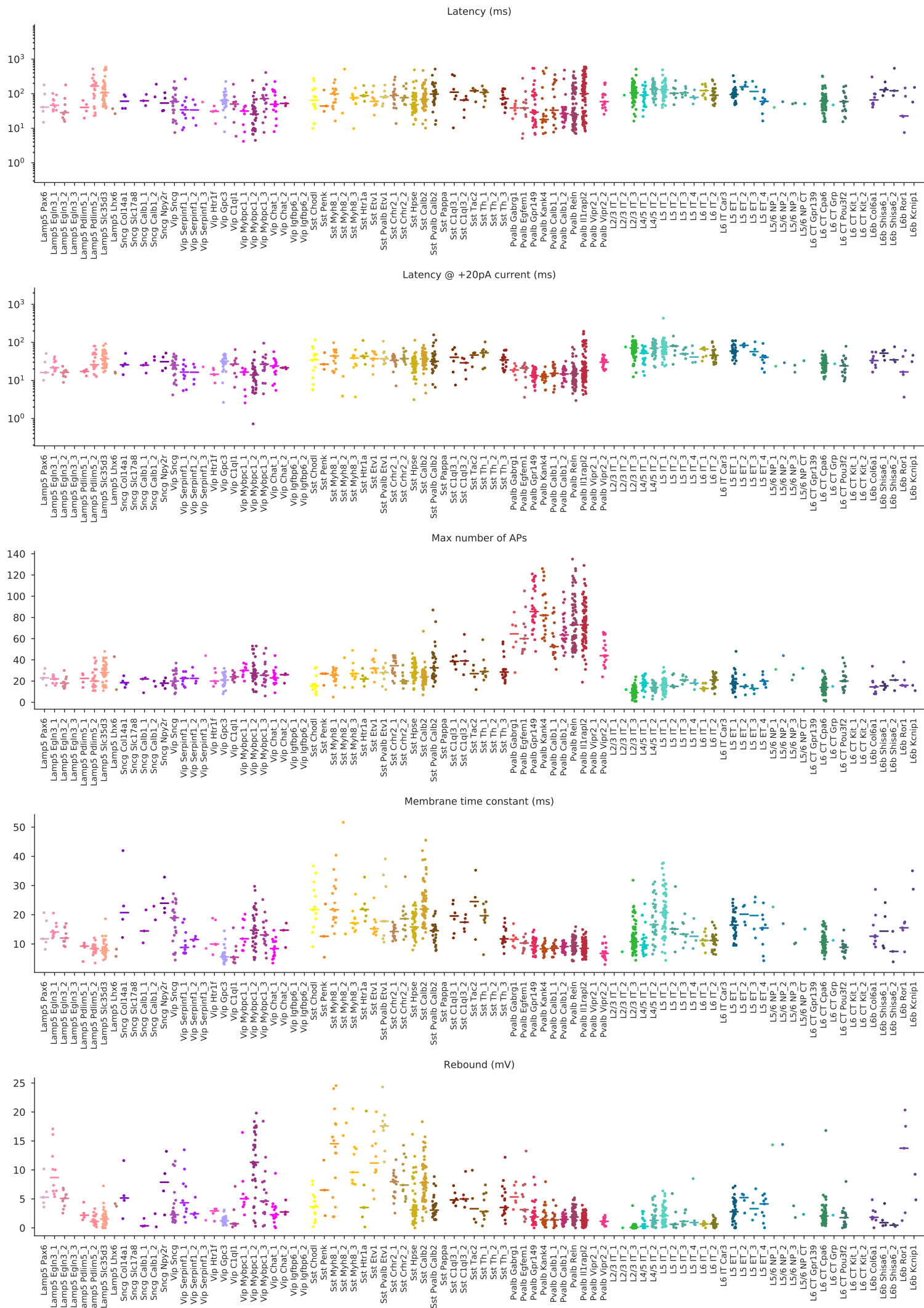

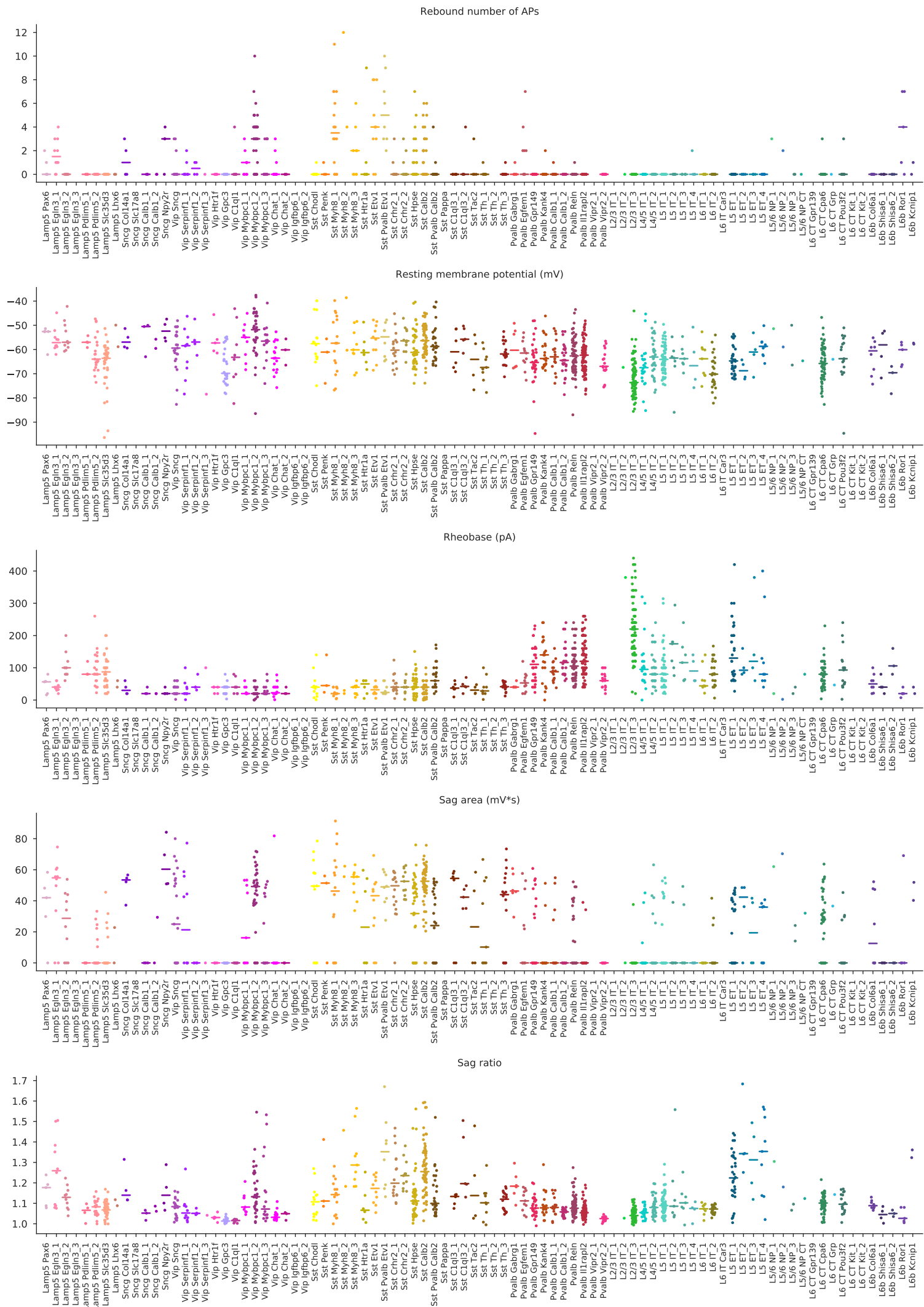

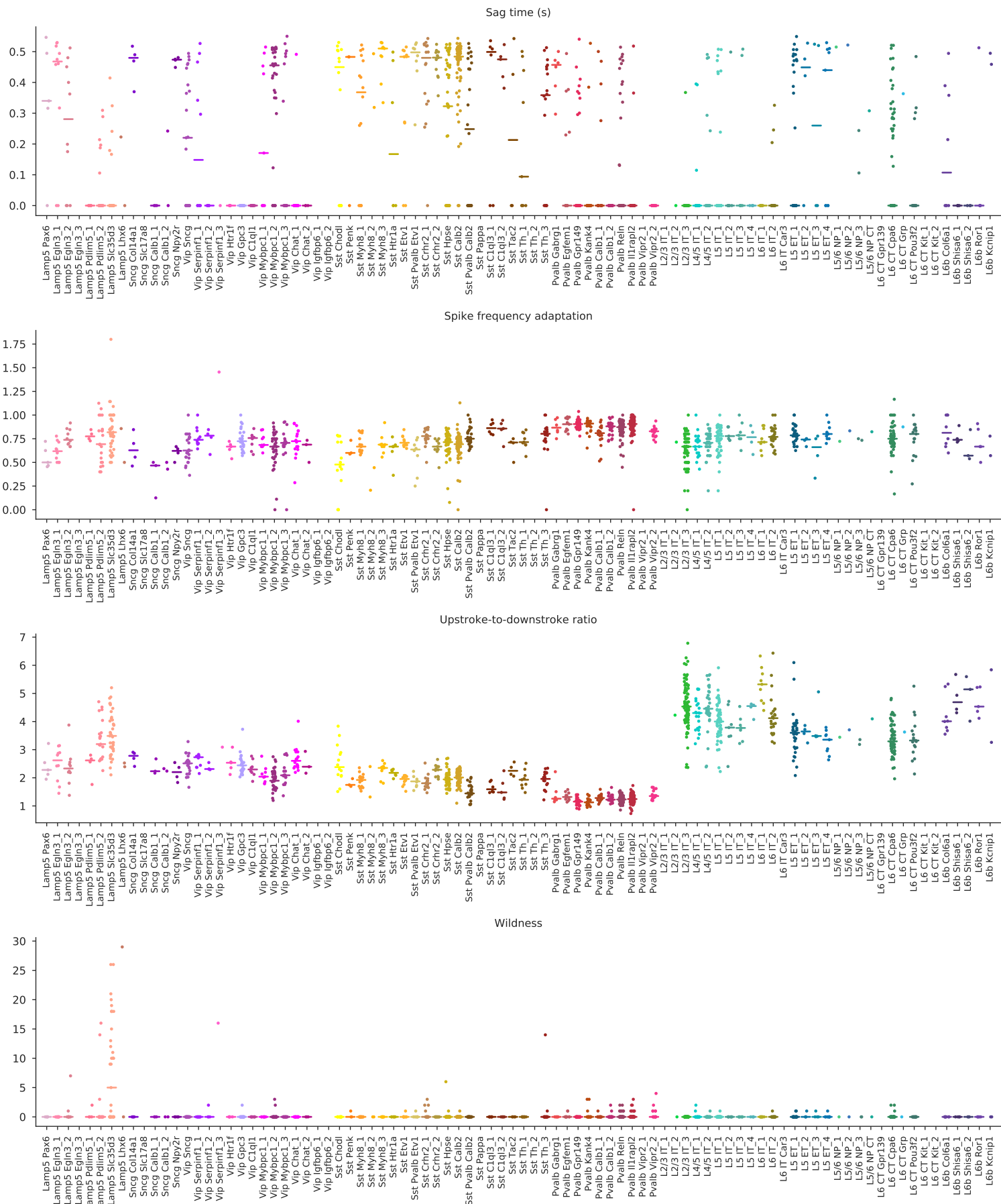

Supplement: Supplementary file 3 — Supplementary File 2: Dot plots of all electrophysiological features shown as in Extended Data Fig. 4. [file 41586_2020_2907_MOESM3_ESM.pdf]
