# Supplementary material for: Phenotypic variation of transcriptomic cell types in mouse motor cortex
Source: Nature. 2020 Nov 12;598(7879):144–50. doi: 10.1038/s41586-020-2907-3 (PMC8113357; doi:10.1038/s41586-020-2907-3)
Supplement: Supplementary file 4 — Supplementary File 3: Full list of computed morphometric statistics. [file 41586_2020_2907_MOESM4_ESM.pdf]

**Somatic Features**	
Normalized soma depth	Distance from pia to soma divided by the distance from pia to white matter (pia=0, wm=1).
Soma radius	Radius of the sphere that approximates the soma (μm).
**Shape Features**	
Defined for the axon, dendrites, and apical dendrites	
Height	Total extent in the z direction (μm).
Robust height	The distance between the 2.5th and 97.5th percentiles of the z coordinates across the whole point cloud (μm).
Width	Total extent in the x direction (μm).
Robust width	The distance between the 2.5th and 97.5th percentiles of the x coordinates across the whole point cloud (μm).
Total length	Total path length across all neurites (μm).
Branch points	Total number of bifurcations.
Defined for the axon and dendrites only	
First bifurcation moment	The average bifurcation position in the z direction, relative to the soma (μm).
Bifurcation standard deviation	The standard deviation of the bifurcation positions in the z direction (μm).
Max branch order	The maximum number of bifurcations passed when tracing a neurite from the tip back to the soma. Branch ordering starts with the soma having branch order 0 and each subsequent bifurcation increases the order by 1.
Tips	The number of end-points.
Max Euclidean distance to soma	Euclidean distance between the soma and the most distal node (μm).
Max path distance to soma	Total path length of the longest neurite from its tip to the soma (μm).
Max segment length	Path length of the longest segment between the two neighbouring bifurcations (μm).
Mean neurite radius	The average radius across all neurites (μm).
Fraction above soma	Fraction of nodes above the soma, i.e. closer to the pia.
X-bias	The absolute difference between x-extents to the left and to the right of the soma (μm).
Z-bias	The difference between z-extents above and below the soma (μm).
Max/Mean/Min branch angle	Maximal/average/minimal branch angle at each bifurcation. A branch angle denotes the angle between the subsequent neurites at a furcation point.
Log max/median/min tortuosity	Log-transformed 99.5th percentile/median/minimal tortuosity across all segments. Tortuosity describes the "bendiness" of a segment and is defined as the ratio of the segment path length to the Euclidean distance between its ends.
Max/Median path angle	99.5th percentile and median across all path angles. A path angle describes the angle between two consecutive sub-segments (1-micron-long each) along the path between the two adjacent bifurcation points. Can take values in the $[0, 180)$ degree range.
Defined for dendrites only	
Stems	Number of neurites extending directly from the soma.
Stems exiting up/down/to the sides	Fraction of stems exiting into the three directions. The direction of a stem is determined by its angle w.r.t. the normal pointing towards the pia. Up: below 45 degrees, down: above 135 degrees, sides: 45–135 degrees.
Defined for the apical dendrite only	
Mean bifurcation distance	The average position of bifurcations projected onto a line connecting the soma to the furthest node, normalized by the total path length. The furthest node is defined in terms of the path length. Takes values in the $(0, 1)$ range.
Bifurcation distance standard deviation	Standard deviation of the normalized bifurcation positions (see above).
Log1p number of outer bifurcations	The number of bifurcations with Euclidean distance to the soma above 0.5 of the maximal Euclidean distance to the soma. This feature was $\log(x+1)$-transformed.
Defined for axon only	
Mean initial segment radius	The average radius of the first axonal segment leaving the soma (μm).
**Overlap Features (only inhibitory cells)**	
EMD axon dendrite	Earth mover's distance (also known as the Wasserstein distance) calculated between the normalized z-profile 20-bin histograms of axon and dendrite. We used the implementation from the `scipy` library.
Log1p fraction of axon above/below dendrite	Fraction of axonal nodes above/below the full z-extent of the dendrites. This feature was $\log(x+1)$-transformed.
Log1p fraction of dendrite above/below axon	(see above).
**Excluded Features**	
Based on small coefficient of variation: $CV < .25$	
Excitatory cells	dendrite above soma, dendrite max branch angle, dendrite max path angle, dendrite mean branch angle, dendrite log median tortuosity
Inhibitory cells	axon max/mean branch angle, axon max/median path angle, dendrite mean branch angle, dendrite max/median path angle, axon log max/median tortuosity
Based on visual inspection	
Inhibitory cells	Log1p fraction of dendrite below axon, dendrite min/max branch angle
