# Supplementary material for: Phenotypic variation of transcriptomic cell types in mouse motor cortex
Source: Nature. Author manuscript; Available in PMC 2021 Oct 8. (PMC8113357; doi:10.1038/s41586-020-2907-3)
Supplement: Supplementary file 1 [file NIHMS1681219-supplement-1681219_SuppFile1.pdf]

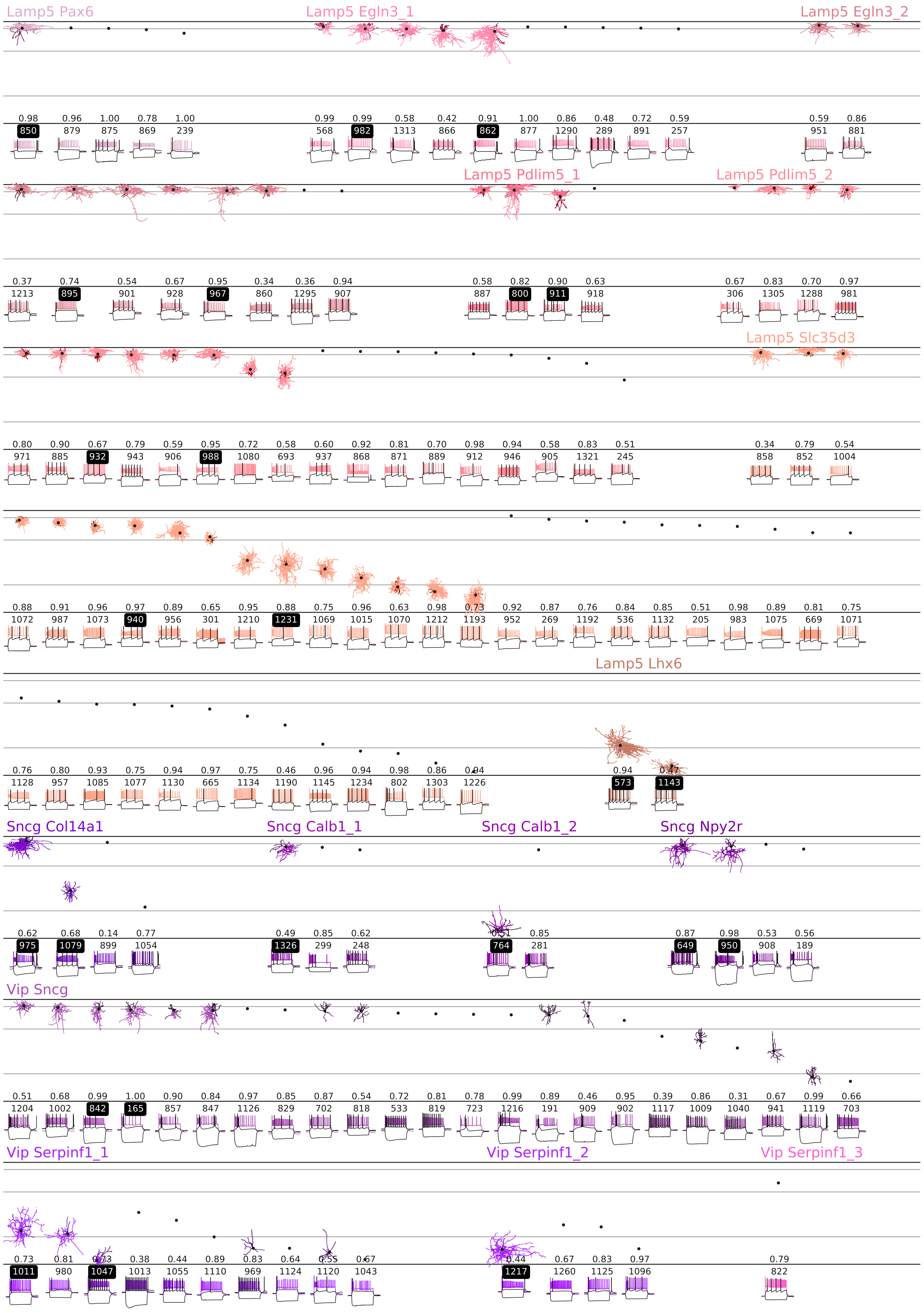

Lamp5 Pax6
Lamp5 Egln3_1
Lamp5 Egln3_2
0.98 0.96 1.00 0.78 1.00
850 879 875 869 239
0.99 0.99 0.58 0.42 0.91 1.00 0.86 0.48 0.72 0.59
568 982 1313 866 862 877 1290 289 891 257
0.59 0.86
951 881
Lamp5 Pdlim5_1
Lamp5 Pdlim5_2
0.37 0.74 0.54 0.67 0.95 0.34 0.36 0.94
1213 895 901 928 967 860 1295 907
0.58 0.82 0.90 0.63
887 800 911 918
0.67 0.83 0.70 0.97
306 1305 1288 981
Lamp5 Slc35d3
0.80 0.90 0.67 0.79 0.59 0.95 0.72 0.58 0.60 0.92 0.81 0.70 0.98 0.94 0.58 0.83 0.51
971 885 932 943 906 988 1080 693 937 868 871 889 912 946 905 1321 245
0.34 0.79 0.54
858 852 1004
0.88 0.91 0.96 0.97 0.89 0.65 0.95 0.88 0.75 0.96 0.63 0.98 0.73 0.92 0.87 0.76 0.84 0.85 0.51 0.98 0.89 0.81 0.75
1072 987 1073 940 956 301 1210 1231 1069 1015 1070 1212 1193 952 269 1192 536 1132 205 983 1075 669 1071
Lamp5 Lhx6
0.76 0.80 0.93 0.75 0.94 0.97 0.75 0.46 0.96 0.94 0.98 0.86 0.94
1128 957 1085 1077 1130 665 1134 1190 1145 1234 802 1303 1226
0.94 0.47
573 1143
Sncg Col14a1
Sncg Calb1_1
Sncg Calb1_2
Sncg Npy2r
0.62 0.68 0.14 0.77
975 1079 899 1054
0.49 0.85 0.62
1326 299 248
0.51 0.85
764 281
0.87 0.98 0.53 0.56
649 950 908 189
Vip Sncg
0.51 0.68 0.99 1.00 0.90 0.84 0.97 0.85 0.87 0.54 0.72 0.81 0.78 0.99 0.89 0.46 0.95 0.39 0.86 0.31 0.67 0.99 0.66
1204 1002 842 165 857 847 1126 829 702 818 533 819 723 1216 191 909 902 1117 1009 1040 941 1119 703
Vip Serpinf1_1
Vip Serpinf1_2
Vip Serpinf1_3
0.73 0.81 0.73 0.38 0.44 0.89 0.83 0.64 0.55 0.67
1011 980 1047 1013 1055 1110 969 1124 1120 1043
0.44 0.67 0.83 0.97
1217 1260 1125 1096
0.79
822

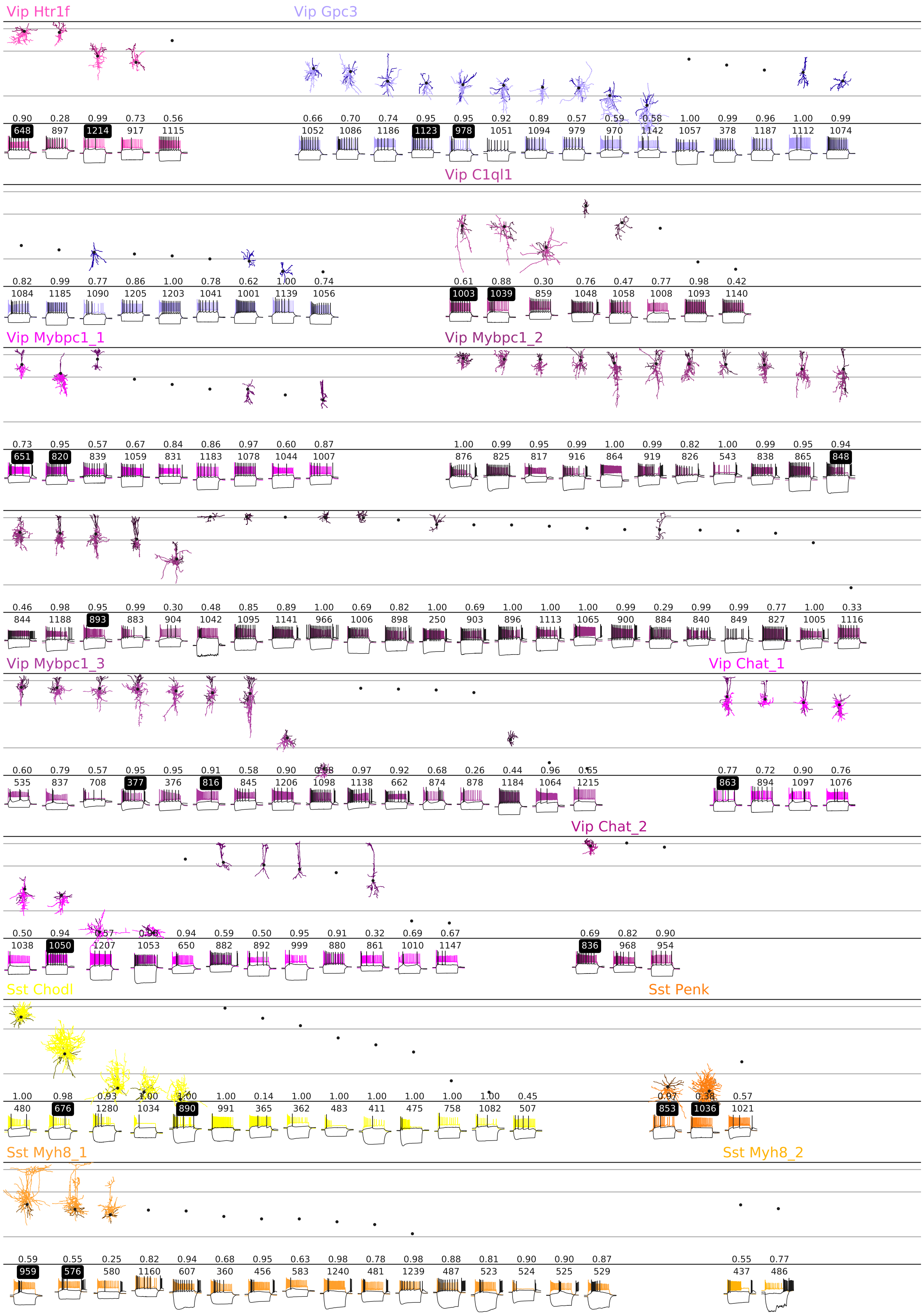

Vip Htr1f
Vip Gpc3
Vip C1ql1
Vip Mybpc1_1
Vip Mybpc1_2
Vip Mybpc1_3
Vip Chat_1
Vip Chat_2
Sst Chodl
Sst Penk
Sst Myh8_1
Sst Myh8_2

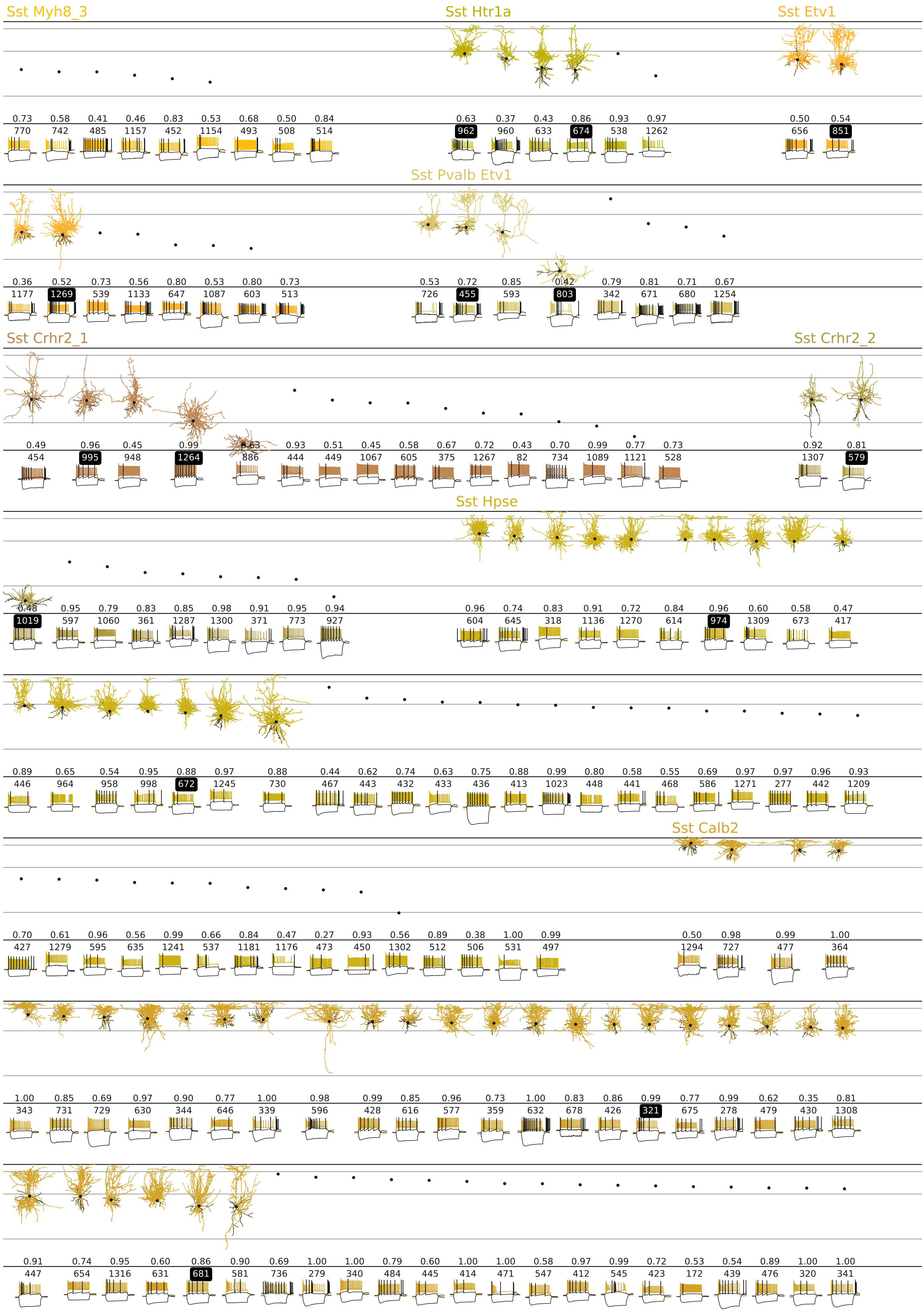
Sst Myh8_3
Sst Htr1a
Sst Etv1
0.73 0.58 0.41 0.46 0.83 0.53 0.68 0.50 0.84 0.63 0.37 0.43 0.86 0.93 0.97 0.50 0.54
770 742 485 1157 452 1154 493 508 514 962 960 633 674 538 1262 656 851
Sst Pvalb Etv1
0.36 0.52 0.73 0.56 0.80 0.53 0.80 0.73 0.53 0.72 0.85 0.42 0.79 0.81 0.71 0.67
1177 1269 539 1133 647 1087 603 513 726 455 593 803 342 671 680 1254
Sst Crhr2_1
Sst Crhr2_2
0.49 0.96 0.45 0.99 0.63 0.93 0.51 0.45 0.58 0.67 0.72 0.43 0.70 0.99 0.77 0.73 0.92 0.81
454 995 948 1264 886 444 449 1067 605 375 1267 82 734 1089 1121 528 1307 579
Sst Hpse
0.48 0.95 0.79 0.83 0.85 0.98 0.91 0.95 0.94 0.96 0.74 0.83 0.91 0.72 0.84 0.96 0.60 0.58 0.47
1019 597 1060 361 1287 1300 371 773 927 604 645 318 1136 1270 614 974 1309 673 417
0.89 0.65 0.54 0.95 0.88 0.97 0.88 0.44 0.62 0.74 0.63 0.75 0.88 0.99 0.80 0.58 0.55 0.69 0.97 0.97 0.96 0.93
446 964 958 998 672 1245 730 467 443 432 433 436 413 1023 448 441 468 586 1271 277 442 1209
Sst Calb2
0.70 0.61 0.96 0.56 0.99 0.66 0.84 0.47 0.27 0.93 0.56 0.89 0.38 1.00 0.99 0.50 0.98 0.99 1.00
427 1279 595 635 1241 537 1181 1176 473 450 1302 512 506 531 497 1294 727 477 364
1.00 0.85 0.69 0.97 0.90 0.77 1.00 0.98 0.99 0.85 0.96 0.73 1.00 0.83 0.86 0.99 0.77 0.99 0.62 0.35 0.81
343 731 729 630 344 646 339 596 428 616 577 359 632 678 426 321 675 278 479 430 1308
0.91 0.74 0.95 0.60 0.86 0.90 0.69 1.00 1.00 0.79 0.60 1.00 1.00 0.58 0.97 0.99 0.72 0.53 0.54 0.89 1.00 1.00
447 654 1316 631 681 581 736 279 340 484 445 414 471 547 412 545 423 172 439 476 320 341

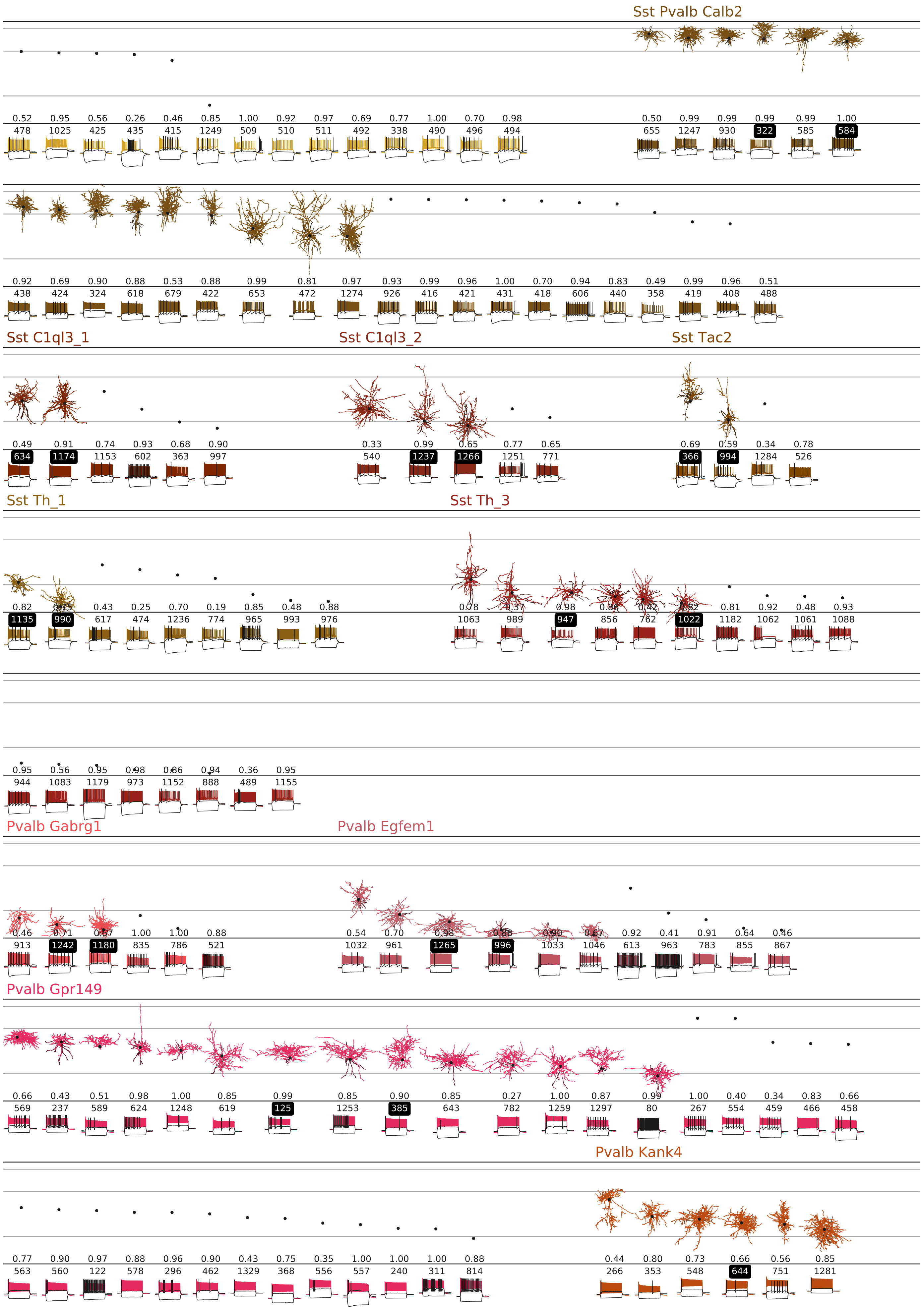

Sst Pvalb Calb2
0.52 0.95 0.56 0.26 0.46 0.85 1.00 0.92 0.97 0.69 0.77 1.00 0.70 0.98 0.50 0.99 0.99 0.99 0.99 1.00
478 1025 425 435 415 1249 509 510 511 492 338 490 496 494 655 1247 930 322 585 584
0.92 0.69 0.90 0.88 0.53 0.88 0.99 0.81 0.97 0.93 0.99 0.96 1.00 0.70 0.94 0.83 0.49 0.99 0.96 0.51
438 424 324 618 679 422 653 472 1274 926 416 421 431 418 606 440 358 419 408 488
Sst C1ql3_1
Sst C1ql3_2
Sst Tac2
0.49 0.91 0.74 0.93 0.68 0.90 0.33 0.99 0.65 0.77 0.65 0.69 0.59 0.34 0.78
634 1174 1153 602 363 997 540 1237 1266 1251 771 366 994 1284 526
Sst Th_1
Sst Th_3
0.82 0.75 0.43 0.25 0.70 0.19 0.85 0.48 0.88 0.78 0.37 0.98 0.86 0.42 0.82 0.81 0.92 0.48 0.93
1135 990 617 474 1236 774 965 993 976 1063 989 947 856 762 1022 1182 1062 1061 1088
0.95 0.56 0.95 0.98 0.86 0.94 0.36 0.95
944 1083 1179 973 1152 888 489 1155
Pvalb Gabrg1
Pvalb Egfem1
0.46 0.71 0.57 1.00 1.00 0.88 0.54 0.70 0.98 0.58 0.90 0.67 0.92 0.41 0.91 0.64 0.46
913 1242 1180 835 786 521 1032 961 1265 996 1033 1046 613 963 783 855 867
Pvalb Gpr149
0.66 0.43 0.51 0.98 1.00 0.85 0.99 0.85 0.90 0.85 0.27 1.00 0.87 0.99 1.00 0.40 0.34 0.83 0.66
569 237 589 624 1248 619 125 1253 385 643 782 1259 1297 80 267 554 459 466 458
Pvalb Kank4
0.77 0.90 0.97 0.88 0.96 0.90 0.43 0.75 0.35 1.00 1.00 1.00 0.88 0.44 0.80 0.73 0.66 0.56 0.85
563 560 122 578 296 462 1329 368 556 557 240 311 814 266 353 548 644 751 1281

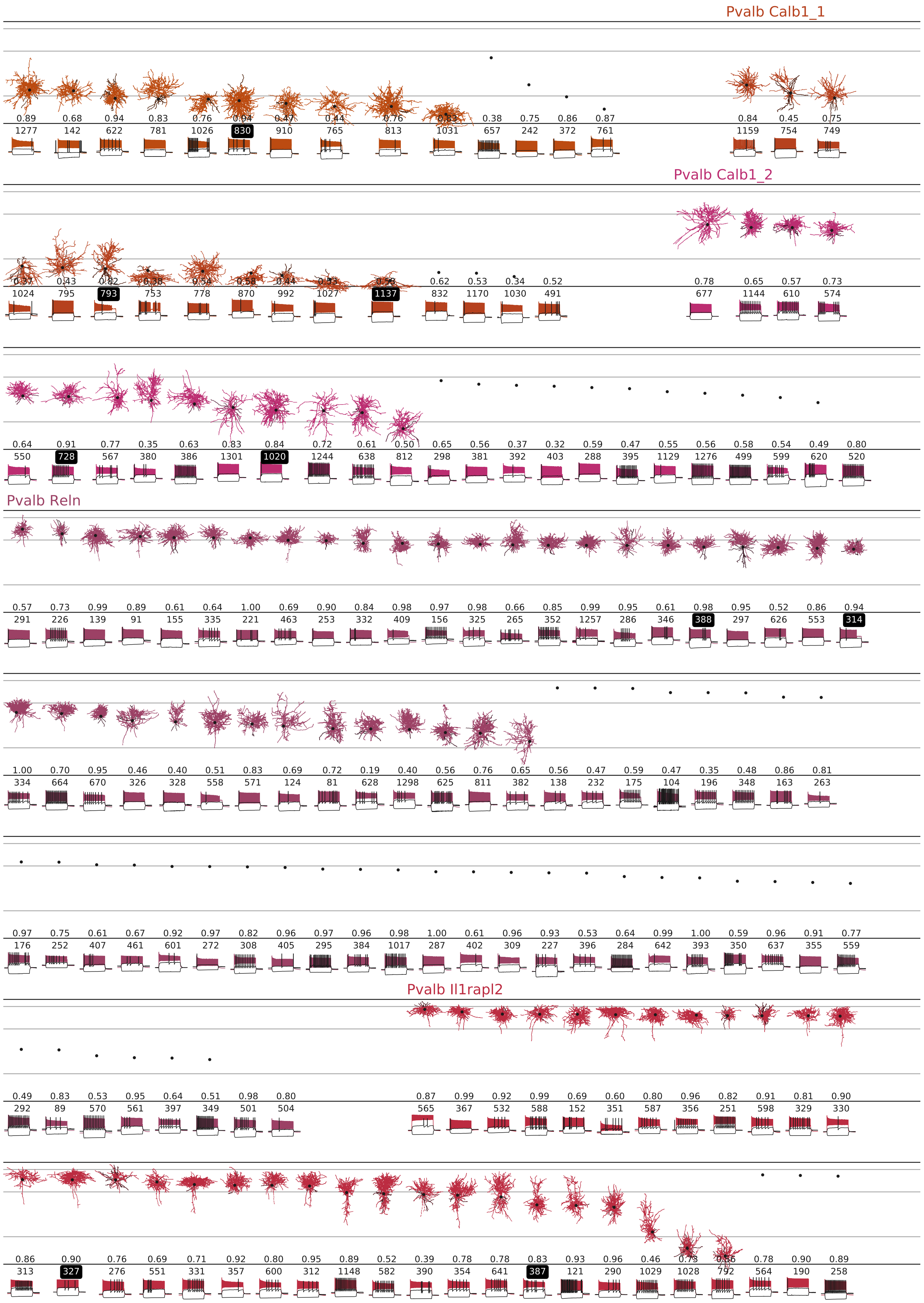

Pvalb Calb1_1
0.89 0.68 0.94 0.83 0.76 0.94 0.47 0.44 0.76 0.83 0.38 0.75 0.86 0.87 0.84 0.45 0.75
1277 142 622 781 1026 830 910 765 813 1031 657 242 372 761 1159 754 749
Pvalb Calb1_2
0.37 0.43 0.82 0.38 0.54 0.58 0.44 0.33 0.78 0.62 0.53 0.34 0.52 0.78 0.65 0.57 0.73
1024 795 793 753 778 870 992 1027 1137 832 1170 1030 491 677 1144 610 574
0.64 0.91 0.77 0.35 0.63 0.83 0.84 0.72 0.61 0.50 0.65 0.56 0.37 0.32 0.59 0.47 0.55 0.56 0.58 0.54 0.49 0.80
550 728 567 380 386 1301 1020 1244 638 812 298 381 392 403 288 395 1129 1276 499 599 620 520
Pvalb Reln
0.57 0.73 0.99 0.89 0.61 0.64 1.00 0.69 0.90 0.84 0.98 0.97 0.98 0.66 0.85 0.99 0.95 0.61 0.98 0.95 0.52 0.86 0.94
291 226 139 91 155 335 221 463 253 332 409 156 325 265 352 1257 286 346 388 297 626 553 314
1.00 0.70 0.95 0.46 0.40 0.51 0.83 0.69 0.72 0.19 0.40 0.56 0.76 0.65 0.56 0.47 0.59 0.47 0.35 0.48 0.86 0.81
334 664 670 326 328 558 571 124 81 628 1298 625 811 382 138 232 175 104 196 348 163 263
0.97 0.75 0.61 0.67 0.92 0.97 0.82 0.96 0.97 0.96 0.98 1.00 0.61 0.96 0.93 0.53 0.64 0.99 1.00 0.59 0.96 0.91 0.77
176 252 407 461 601 272 308 405 295 384 1017 287 402 309 227 396 284 642 393 350 637 355 559
Pvalb Il1rapl2
0.49 0.83 0.53 0.95 0.64 0.51 0.98 0.80 0.87 0.99 0.92 0.99 0.69 0.60 0.80 0.96 0.82 0.91 0.81 0.90
292 89 570 561 397 349 501 504 565 367 532 588 152 351 587 356 251 598 329 330
0.86 0.90 0.76 0.69 0.71 0.92 0.80 0.95 0.89 0.52 0.39 0.78 0.78 0.83 0.93 0.96 0.46 0.73 0.56 0.78 0.90 0.89
313 327 276 551 331 357 600 312 1148 582 390 354 641 387 121 290 1029 1028 792 564 190 258

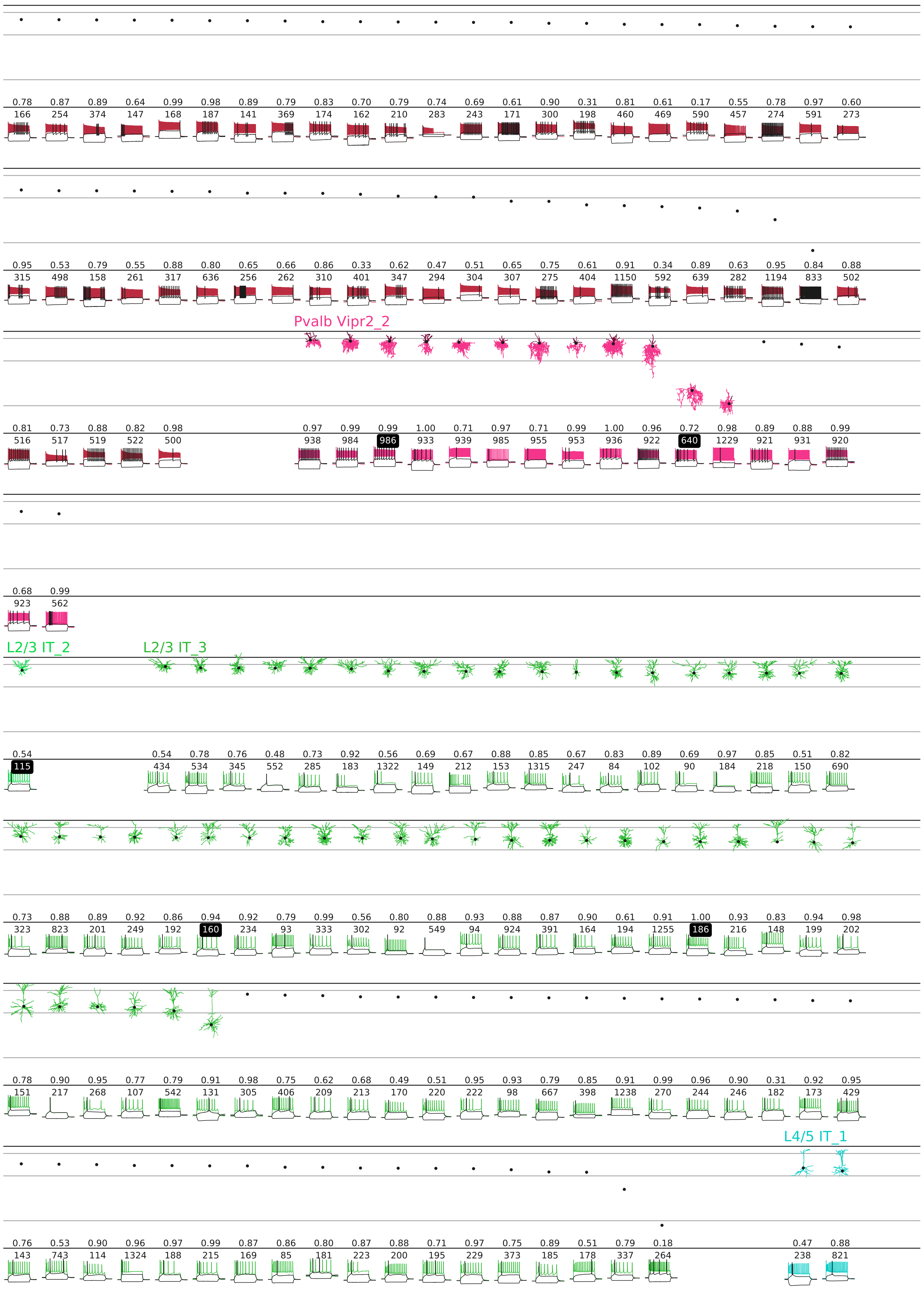

0.78 0.87 0.89 0.64 0.99 0.98 0.89 0.79 0.83 0.70 0.79 0.74 0.69 0.61 0.90 0.31 0.81 0.61 0.17 0.55 0.78 0.97 0.60
166 254 374 147 168 187 141 369 174 162 210 283 243 171 300 198 460 469 590 457 274 591 273
0.95 0.53 0.79 0.55 0.88 0.80 0.65 0.66 0.86 0.33 0.62 0.47 0.51 0.65 0.75 0.61 0.91 0.34 0.89 0.63 0.95 0.84 0.88
315 498 158 261 317 636 256 262 310 401 347 294 304 307 275 404 1150 592 639 282 1194 833 502
Pvalb Vipr2_2
0.81 0.73 0.88 0.82 0.98 0.97 0.99 0.99 1.00 0.71 0.97 0.71 0.99 1.00 0.96 0.72 0.98 0.89 0.88 0.99
516 517 519 522 500 938 984 986 933 939 985 955 953 936 922 640 1229 921 931 920
0.68 0.99
923 562
L2/3 IT_2
L2/3 IT_3
0.54 0.54 0.78 0.76 0.48 0.73 0.92 0.56 0.69 0.67 0.88 0.85 0.67 0.83 0.89 0.69 0.97 0.85 0.51 0.82
115 434 534 345 552 285 183 1322 149 212 153 1315 247 84 102 90 184 218 150 690
0.73 0.88 0.89 0.92 0.86 0.94 0.92 0.79 0.99 0.56 0.80 0.88 0.93 0.88 0.87 0.90 0.61 0.91 1.00 0.93 0.83 0.94 0.98
323 823 201 249 192 160 234 93 333 302 92 549 94 924 391 164 194 1255 186 216 148 199 202
0.78 0.90 0.95 0.77 0.79 0.91 0.98 0.75 0.62 0.68 0.49 0.51 0.95 0.93 0.79 0.85 0.91 0.99 0.96 0.90 0.31 0.92 0.95
151 217 268 107 542 131 305 406 209 213 170 220 222 98 667 398 1238 270 244 246 182 173 429
L4/5 IT_1
0.76 0.53 0.90 0.96 0.97 0.99 0.87 0.86 0.80 0.87 0.88 0.71 0.97 0.75 0.89 0.51 0.79 0.18 0.47 0.88
143 743 114 1324 188 215 169 85 181 223 200 195 229 373 185 178 337 264 238 821

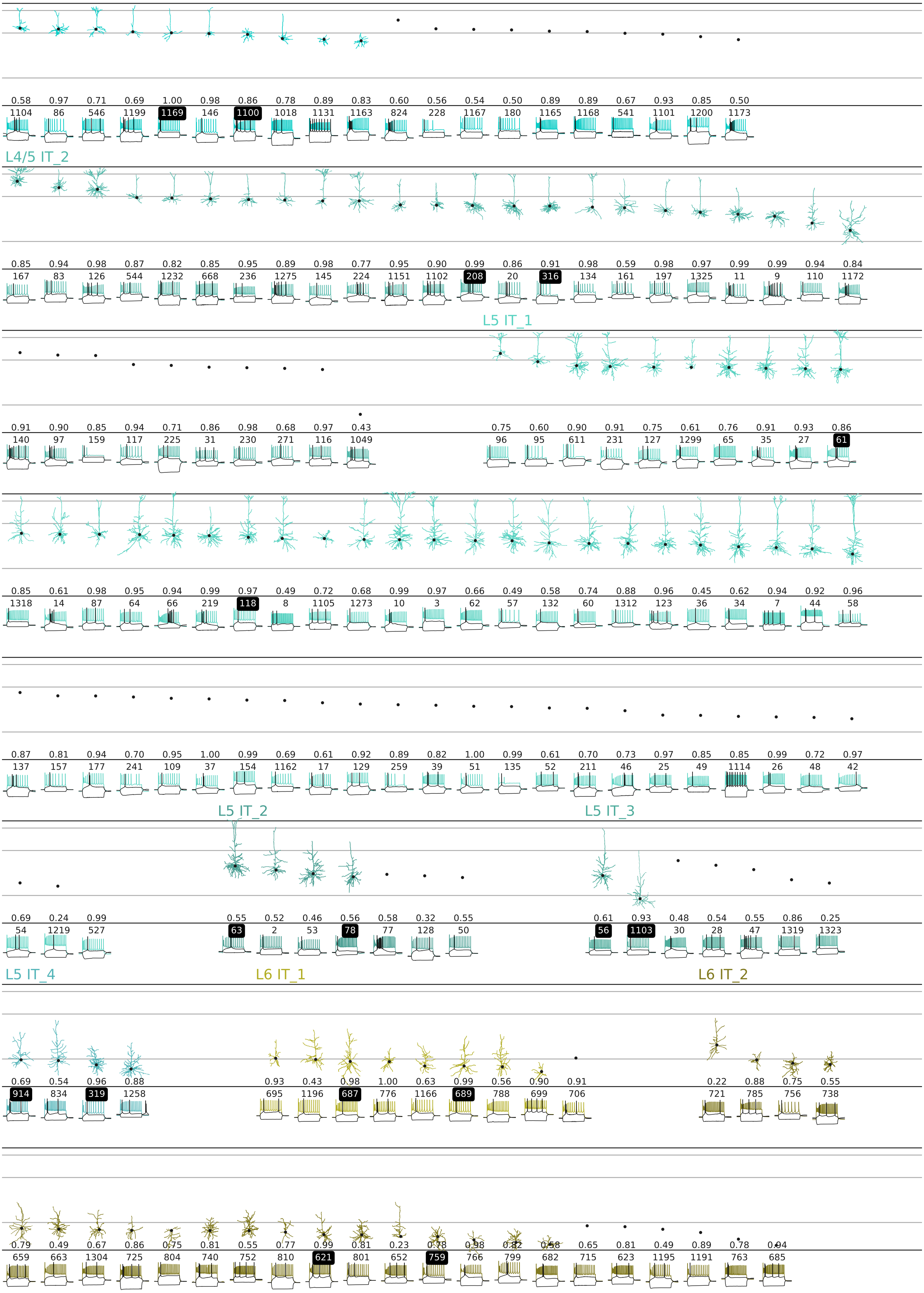

0.58 0.97 0.71 0.69 1.00 0.98 0.86 0.78 0.89 0.83 0.60 0.56 0.54 0.50 0.89 0.89 0.67 0.93 0.85 0.50
1104 86 546 1199 1169 146 1100 1018 1131 1163 824 228 1167 180 1165 1168 541 1101 1200 1173
L4/5 IT_2
0.85 0.94 0.98 0.87 0.82 0.85 0.95 0.89 0.98 0.77 0.95 0.90 0.99 0.86 0.91 0.98 0.59 0.98 0.97 0.99 0.99 0.94 0.84
167 83 126 544 1232 668 236 1275 145 224 1151 1102 208 20 316 134 161 197 1325 11 9 110 1172
L5 IT_1
0.91 0.90 0.85 0.94 0.71 0.86 0.98 0.68 0.97 0.43
140 97 159 117 225 31 230 271 116 1049
0.75 0.60 0.90 0.91 0.75 0.61 0.76 0.91 0.93 0.86
96 95 611 231 127 1299 65 35 27 61
0.85 0.61 0.98 0.95 0.94 0.99 0.97 0.49 0.72 0.68 0.99 0.97 0.66 0.49 0.58 0.74 0.88 0.96 0.45 0.62 0.94 0.92 0.96
1318 14 87 64 66 219 118 8 1105 1273 10 3 62 57 132 60 1312 123 36 34 7 44 58
0.87 0.81 0.94 0.70 0.95 1.00 0.99 0.69 0.61 0.92 0.89 0.82 1.00 0.99 0.61 0.70 0.73 0.97 0.85 0.85 0.99 0.72 0.97
137 157 177 241 109 37 154 1162 17 129 259 39 51 135 52 211 46 25 49 1114 26 48 42
L5 IT_2
L5 IT_3
0.69 0.24 0.99
54 1219 527
0.55 0.52 0.46 0.56 0.58 0.32 0.55
63 2 53 78 77 128 50
0.61 0.93 0.48 0.54 0.55 0.86 0.25
56 1103 30 28 47 1319 1323
L5 IT_4
L6 IT_1
L6 IT_2
0.69 0.54 0.96 0.88
914 834 319 1258
0.93 0.43 0.98 1.00 0.63 0.99 0.56 0.90 0.91
695 1196 687 776 1166 689 788 699 706
0.22 0.88 0.75 0.55
721 785 756 738
0.79 0.49 0.67 0.86 0.75 0.81 0.55 0.77 0.99 0.81 0.23 0.78 0.98 0.82 0.58 0.65 0.81 0.49 0.89 0.78 0.94
659 663 1304 725 804 740 752 810 621 801 652 759 766 799 682 715 623 1195 1191 763 685

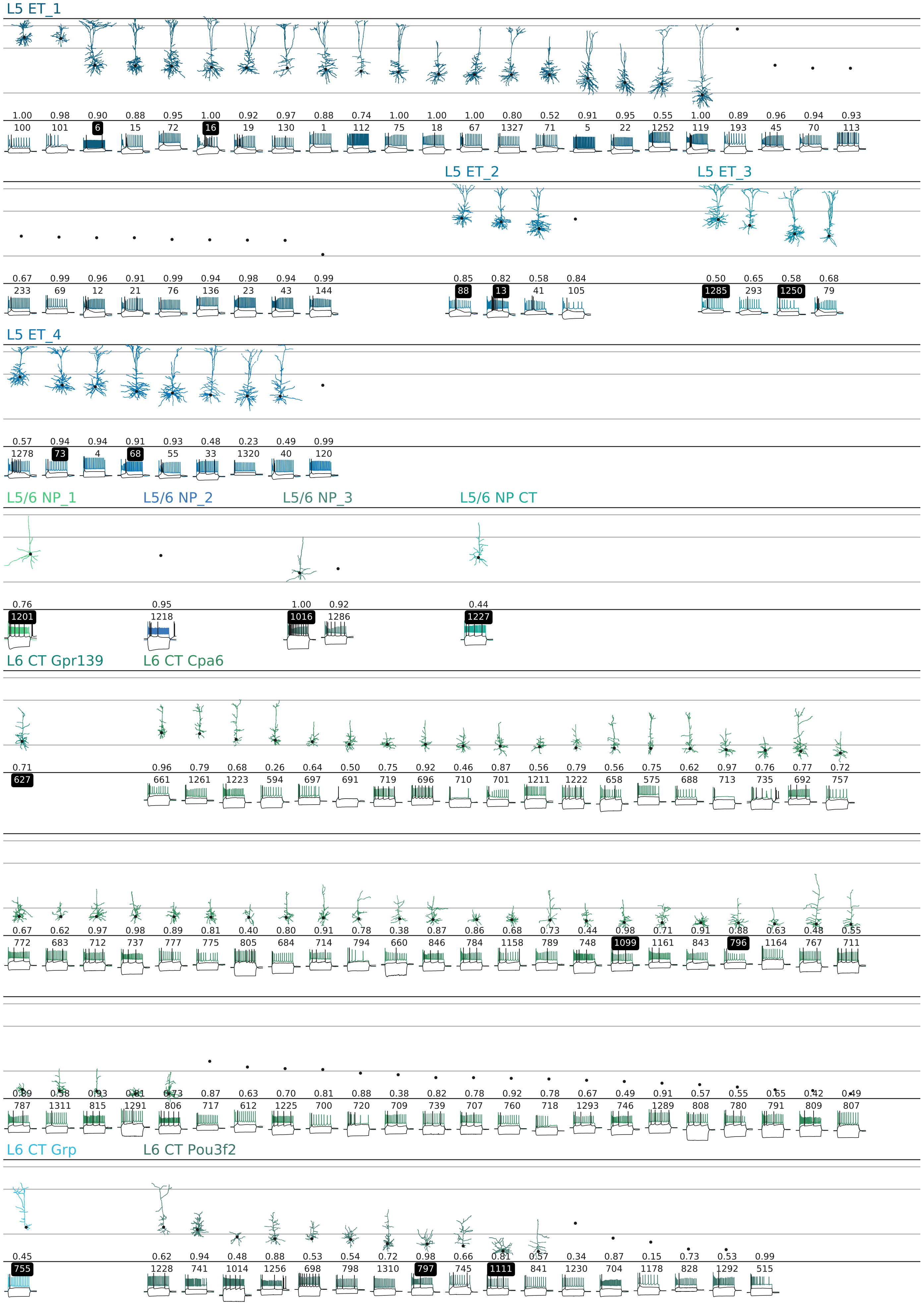
L5 ET_1
1.00 0.98 0.90 0.88 0.95 1.00 0.92 0.97 0.88 0.74 1.00 1.00 1.00 0.80 0.52 0.91 0.95 0.55 1.00 0.89 0.96 0.94 0.93
100 101 6 15 72 16 19 130 1 112 75 18 67 1327 71 5 22 1252 119 193 45 70 113
0.67 0.99 0.96 0.91 0.99 0.94 0.98 0.94 0.99
233 69 12 21 76 136 23 43 144
L5 ET_2
0.85 0.82 0.58 0.84
88 13 41 105
L5 ET_3
0.50 0.65 0.58 0.68
1285 293 1250 79
L5 ET_4
0.57 0.94 0.94 0.91 0.93 0.48 0.23 0.49 0.99
1278 73 4 68 55 33 1320 40 120
L5/6 NP_1
0.76
1201
L5/6 NP_2
0.95
1218
L5/6 NP_3
1.00 0.92
1016 1286
L5/6 NP CT
0.44
1227
L6 CT Gpr139
0.71
627
L6 CT Cpa6
0.96 0.79 0.68 0.26 0.64 0.50 0.75 0.92 0.46 0.87 0.56 0.79 0.56 0.75 0.62 0.97 0.76 0.77 0.72
661 1261 1223 594 697 691 719 696 710 701 1211 1222 658 575 688 713 735 692 757
0.67 0.62 0.97 0.98 0.89 0.81 0.40 0.80 0.91 0.78 0.38 0.87 0.86 0.68 0.73 0.44 0.98 0.71 0.91 0.88 0.63 0.48 0.55
772 683 712 737 777 775 805 684 714 794 660 846 784 1158 789 748 1099 1161 843 796 1164 767 711
0.89 0.58 0.93 0.81 0.73 0.87 0.63 0.70 0.81 0.88 0.38 0.82 0.78 0.92 0.78 0.67 0.49 0.91 0.57 0.55 0.65 0.42 0.49
787 1311 815 1291 806 717 612 1225 700 720 709 739 707 760 718 1293 746 1289 808 780 791 809 807
L6 CT Grp
0.45
755
L6 CT Pou3f2
0.62 0.94 0.48 0.88 0.53 0.54 0.72 0.98 0.66 0.81 0.57 0.34 0.87 0.15 0.73 0.53 0.99
1228 741 1014 1256 698 798 1310 797 745 1111 841 1230 704 1178 828 1292 515

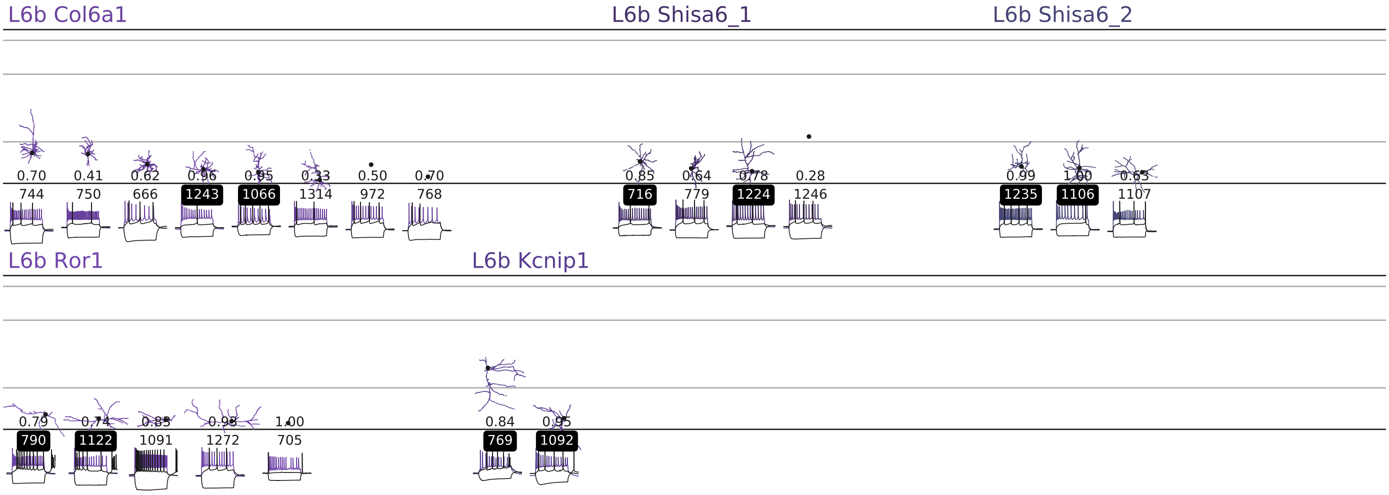
L6b Col6a1
L6b Shisa6_1
L6b Shisa6_2
0.70 0.41 0.62 0.96 0.95 0.33 0.50 0.70
744 750 666 1243 1066 1314 972 768
0.85 0.64 0.78 0.28
716 779 1224 1246
0.99 1.00 0.65
1235 1106 1107
L6b Ror1
L6b Kcnip1
0.79 0.74 0.85 0.98 1.00
790 1122 1091 1272 705
0.84 0.95
769 1092
