# Supplementary material for: Phenotypic variation of transcriptomic cell types in mouse motor cortex
Source: Nature. Author manuscript; Available in PMC 2021 Oct 8. (PMC8113357; doi:10.1038/s41586-020-2907-3)
Supplement: Supplementary file 2 [file NIHMS1681219-supplement-1681219_SuppGuide.pdf]

Supplementary Files

Supplementary File 1: All reconstructed morphologies and electrophysiological traces shown as in Extended Data Fig. 3, sorted by transcriptomic type.

Supplementary File 2: Dot plots of all electrophysiological features shown as in Extended Data Fig. 4.

Supplementary File 3: Full list of computed morphometric statistics.
